# Helicobacter pylori Modulates Heptose Metabolite Biosynthesis and Heptose-Dependent Innate Immune Host Cell Activation by Multiple Mechanisms

**DOI:** 10.1128/spectrum.03132-22

**Published:** 2023-04-27

**Authors:** Martina Hauke, Felix Metz, Johanna Rapp, Larissa Faass, Simon H. Bats, Sandra Radziej, Hannes Link, Wolfgang Eisenreich, Christine Josenhans

**Affiliations:** a Max von Pettenkofer Institute, Ludwig Maximilians University Munich, München, Germany; b Bavarian NMR Center–Structural Membrane Biochemistry, Department of Chemistry, Technical University Munich, Garching, Germany; c Bacterial Metabolomics, CMFI, University Tübingen, Tübingen, Germany; University of North Dakota

**Keywords:** ADP-heptose, ALPK1-TIFA, *Helicobacter pylori*, mass spectrometry, NMR, bacterial metabolites, heptose-monophosphate, innate immunity, lipopolysaccharide

## Abstract

Heptose metabolites including ADP-d-glycero-β-d-manno-heptose (ADP-heptose) are involved in bacterial lipopolysaccharide and cell envelope biosynthesis. Recently, heptoses were also identified to have potent proinflammatory activity on human cells as novel microbe-associated molecular patterns. The gastric pathogenic bacterium Helicobacter pylori produces heptose metabolites, which it transports into human cells through its Cag type 4 secretion system. Using H. pylori as a model, we have addressed the question of how proinflammatory ADP-heptose biosynthesis can be regulated by bacteria. We have characterized the interstrain variability and regulation of heptose biosynthesis genes and the modulation of heptose metabolite production by H. pylori, which impact cell-autonomous proinflammatory human cell activation. HldE, a central enzyme of heptose metabolite biosynthesis, showed strong sequence variability between strains and was also variably expressed between strains. Amounts of gene transcripts in the *hldE* gene cluster displayed intrastrain and interstrain differences, were modulated by host cell contact and the presence of the *cag* pathogenicity island, and were affected by carbon starvation regulator A (CsrA). We reconstituted four steps of the H. pylori lipopolysaccharide (LPS) heptose biosynthetic pathway *in vitro* using recombinant purified GmhA, HldE, and GmhB proteins. On the basis of one- and two-dimensional nuclear magnetic resonance (NMR) spectroscopy and mass spectrometry, the structures of major reaction products were identified as β-d-ADP-heptose and β-heptose-1-monophosphate. A proinflammatory heptose-monophosphate variant was also identified for the first time as a novel cell-active product in H. pylori bacteria. Separate purified HldE subdomains and variant HldE allowed us to uncover additional strain variation in generating heptose metabolites.

**IMPORTANCE** Bacterial heptose metabolites, intermediates of lipopolysaccharide (LPS) biosynthesis, are novel microbe-associated molecular patterns (MAMPs) that activate proinflammatory signaling. In the gastric pathogen Helicobacter pylori, heptoses are transferred into host cells by the Cag type IV secretion system, which is also involved in carcinogenesis. Little is known about how H. pylori, which is highly strain variable, regulates heptose biosynthesis and downstream host cell activation. We report here that the regulation of proinflammatory heptose production by H. pylori is strain specific. Heptose gene cluster activity is modulated by the presence of an active *cag* pathogenicity island (*cag*PAI), contact with human cells, and the carbon starvation regulator A. Reconstitution with purified biosynthesis enzymes and purified bacterial lysates allowed us to biochemically characterize heptose pathway products, identifying a heptose-monophosphate variant as a novel proinflammatory metabolite. These findings emphasize that the bacteria use heptose biosynthesis to fine-tune inflammation and also highlight opportunities to mine the heptose biosynthesis pathway as a potential therapeutic target against infection, inflammation, and cancer.

## INTRODUCTION

Heptose derivatives, including ADP-d-glycero-β-d-manno-heptose (ADP-heptose), are produced by Gram-negative and Gram-positive bacteria as building blocks for the biosynthesis of lipopolysaccharide (LPS) structures and other envelope components, such as surface layers (1). In particular, most Gram-negative bacteria require heptose sugars in the inner core of their LPS. Recently, (ADP)-heptose metabolites were also defined as a class of novel microbe-associated molecular patterns (MAMPs) produced by Gram-negative bacteria. Via pattern recognition, these heptose metabolites, in particular ADP-heptose, can be recognized inside mammalian cells and lead to downstream NF-κB activation through the ALPK1-TIFA axis and the formation of tumor necrosis factor receptor-associated factor (TRAF)-interacting proteins with forkhead-associated domain complexes (TIFAsomes) (2–4). This innate cell activation mechanism was already described for a number of pathogenic bacteria, including Neisseria gonorrhoeae (5, 6), Yersinia enterocolitica (4), diverse Escherichia coli strains (4), Shigella flexneri (2), Campylobacter jejuni (7), and Helicobacter pylori (3, 8–12). While some bacteria release the heptose metabolites directly into the medium (7, 13), other species use the targeted properties of complex bacterial membrane secretion systems to inject some of these metabolites into the cytoplasm of mammalian host cells. For example, H. pylori appears to use mainly its Cag type 4 secretion system (CagT4SS) to also mediate the transfer of heptoses into human gastric epithelial cells (3, 10, 11) and monocytes/macrophages (12), while *Enterobacteriaceae* (*Shigella* and *Yersinia*) and Pseudomonas spp. may also enlist their type 3 secretion systems (T3SS) for a similar purpose (14). Hence, similar to T3SS, which can transport both proteins and metabolites for host cell targeting, this also seems to apply for T4SS.

The core heptose biosynthesis pathway leading to primary production of heptoses is present in most Gram-negative bacteria and also exists in some Gram-positive bacteria (15, 16). Several Gram-negative bacteria use heptoses in their LPS inner core and in their LPS outer core or outer chains (*O*-antigen chains) (1, 17–19). Various bacteria also incorporate heptose sugars into their surface layers or outer polysaccharide capsules (1, 16, 20–24) and into bacterial surface-associated proteins (25). Glycosylation with glycero-manno-heptose was, for instance, detected in the AIDA-I outer membrane-associated autotransporter adhesin of intestinal pathogenic enteroaggregative E. coli (25). The canonical biosynthesis pathway of LPS core heptoses leading to nucleotide (ADP)-activated heptose was first reported in E. coli, Haemophilus influenzae, and Aneurinibacillus thermoaerophilus (15, 16, 26–28). GDP-heptose, in addition to ADP-heptose metabolites, has also been identified, for instance, in Mycobacterium tuberculosis and enteropathogenic Yersinia enterocolitica (29, 30). The canonical heptose biosynthesis pathway generally consists of five steps, which are mediated by the consecutive action of four or five different enzymes, for instance, in E. coli and H. pylori, GmhA, HldE (RfaE), GmhB, and HldD (RfaD) (16, 31, 32). ADP-activated heptoses, delivered by the last biosynthesis steps, can then serve as substrates for the downstream glycosyltransferases involved, for example, in the assembly of the LPS inner core oligosaccharides (33, 34).

Despite the long-standing knowledge about the canonical heptose biosynthesis pathway, very little is known about the regulation of the biosynthetic gene cluster, the diverse enzymatic activity profiles of the contributing enzymes, and the roles of various heptose biosynthesis genes in different bacteria and strain variability of those traits in different bacterial species. The gene cluster and single gene or enzyme activities have been characterized most intensively in E. coli (15, 28, 31, 32, 35). H. pylori, the model organism that is studied here, contains short heptane chains with α-configured d-glycero-d-manno-heptose units in its LPS core (19, 36). In addition, some, preferentially non-Asian strains assemble branched heptose subunits in their outer core (19). H. pylori harbors one single canonical heptose biosynthesis gene cluster, consisting of *gmhA*/*rfaA* (HP0857), a bifunctional bidomain-encoding *hldE*/*rfaE* gene (HP0858), as in E. coli, and *gmhB* (HP0860) and *hldD* genes (HP0859). In H. pylori, the individual enzymes encoded in the core heptose biosynthesis cluster were putatively assigned, and the enzymatic activities of these proteins were partially reported (8, 37). d-Glycero-β-d-manno-heptose-1,7-bisphosphate (β-HBP), a major reaction product in *Neisseria* (5), and ADP-heptose, produced by H. pylori, Y. enterocolitica, Salmonella, and E. coli, were shown to exhibit an innate activation potential toward human cells via the ALKP1-TIFA axis (4, 8).

In the case of guided host cell activation by the secretion system of pathogenic bacteria (for example, by transport or translocation of heptose metabolites into human cells), the important question arises of whether and how the bacteria have developed ways to cell contact-dependently and strain-dependently control the biosynthesis of phospho-heptose derivatives and their innate activity on target cells. To address these open questions, we have studied H. pylori, which directs heptose metabolites into human cells mainly by means of its T4SS (3, 11, 12). First, we tested the regulation of genes in the heptose biosynthesis cluster in various strains and assessed the influence of several physiological parameters on the regulation of heptose biosynthesis in H. pylori. Using this approach, we have identified that strain identity, the presence of a functional CagT4SS, or the allelic diversity of the biosynthesis genes themselves contributed to strain- and condition-dependent modulation of heptose biosynthesis and metabolite activity on host cells. By reconstituting the pathway *in vitro* and characterizing final and intermediate products *in vitro* and in H. pylori lysates biochemically, we have also gained insight into the variation of known and novel output metabolites (products) of the heptose pathway and their activity as novel MAMPs.

## RESULTS

### Strain-specific sequence diversity, differential regulation of genes, and protein expression in the H. pylori LPS heptose biosynthesis gene cluster.

**FIG 1:**
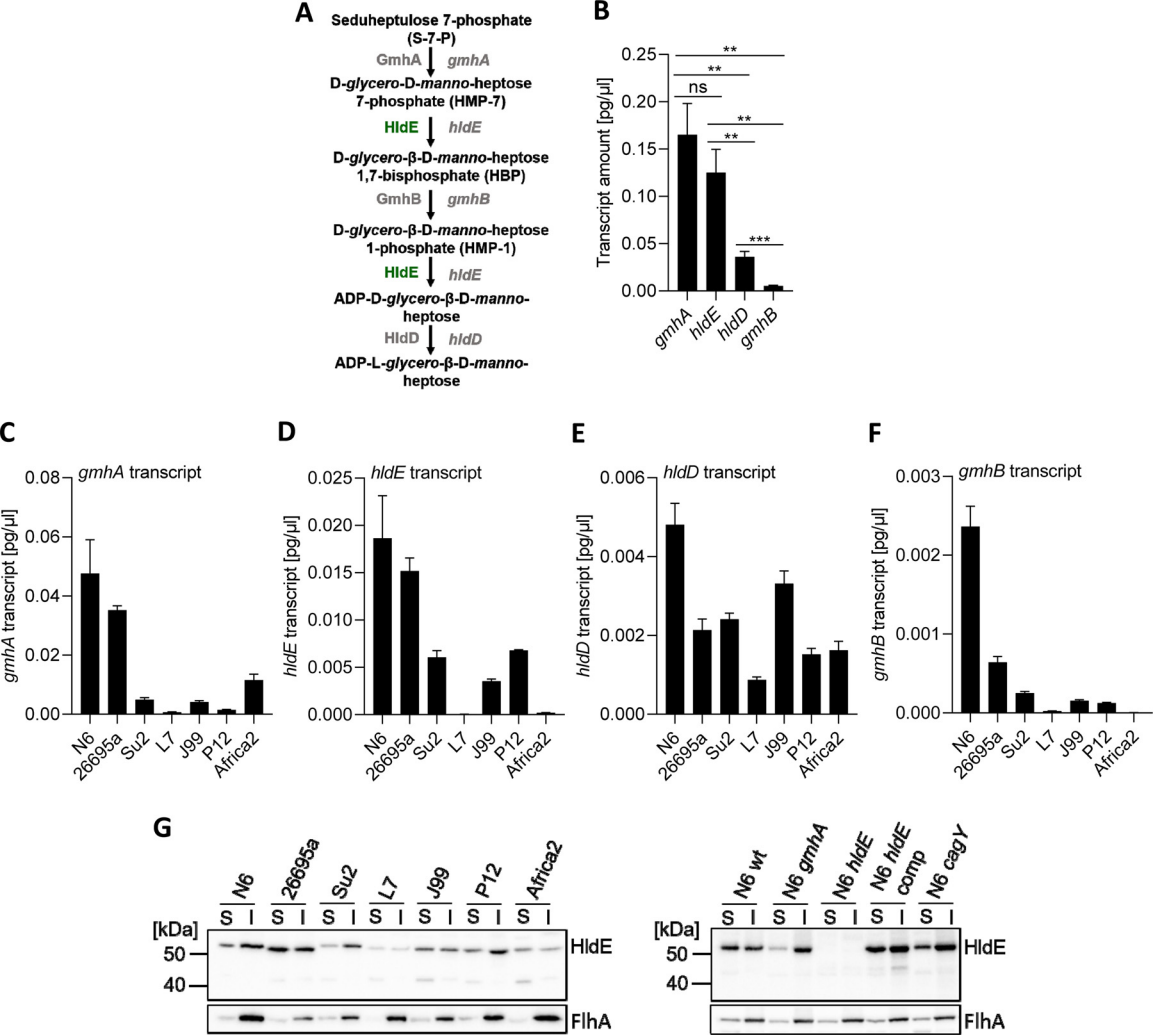
Expression of heptose biosynthesis cluster genes and gene product HldE in H. pylori is gene specific and strain specific. (A) Schematic of the H. pylori 5-step ADP-l-heptose biosynthesis pathway with enzymes and proposed intermediate reaction products. The central enzyme HldE/RfaE (green) is encoded by the gene HP0858 (*hldE*) and is a bifunctional enzyme performing two steps of the synthesis cascade. (B) Quantification of absolute transcript amounts of each transcript of heptose biosynthesis cluster genes *gmhA*, *hldE* (HP0858), *hldD*, and *gmhB* on H. pylori strain N6 cDNA, as determined by RT-qPCR; ns, not significant. (C to F) Diverse H. pylori wild-type strains of different geographical origins were used to compare the transcript amounts of genes *gmhA* (C), *hldE* (D), (*hldD*/*rfaD*) (E), and (*gmhB*) (F) between different strains. All RT-qPCR assays were performed in technical triplicates. The results are given as absolute values for transcript amounts normalized with a correction factor to 16S rRNA transcript amounts for each strain. Strain N6 was used as a reference for normalization. (G) Immunoblot (anti-HldE antiserum, 1:20,000) detecting the bifunctional protein HldE and the loading and fractionation control protein FlhA (flagellar membrane protein) in soluble (S) and insoluble (I) fractions of different H. pylori wild-type strains (left) and of selected isogenic mutants (right) of the H. pylori strain N6. Ten micrograms of total protein was loaded in each lane to provide equalized amounts; wt, wild-type. Comparisons for statistically significant differences in B were performed between all conditions using an unpaired Student’s *t* test; significant *P* values are marked with asterisks; **, *P* < 0.01; ***, *P* < 0.001. Statistics for all pairwise comparisons in C to F were performed by two-way analysis of variance (ANOVA), followed by a Tukey *post hoc* test and are summarized in Table S1 in the supplemental material.

To form a basis for assessing the heptose biosynthesis capacities in H. pylori and their strain-dependent genetic diversity, we initially analyzed the genomic organization of the heptose biosynthesis gene cluster and sequence polymorphisms of its cluster genes in different H. pylori strains. The H. pylori heptose biosynthesis gene cluster, similar to the C. jejuni cluster (7), has a counterintuitive organization, which is conserved in all H. pylori strains; the genes that are relevant in the later steps of the biosynthesis pathway are transcribed first and are located directly downstream of the nontranslated 3′ region. The *gmhA* gene (HP0857), which encodes the first enzyme in the pathway, is the last gene of the cluster (Fig. S1A in the supplemental material). *hldE* (HP0858), the second to last gene in the operon, encodes a bifunctional enzyme with two domains (d1 and d2) that are required for two separate steps in the biosynthesis pathway (Fig. 1A; Fig. S1A). Interestingly, the H. pylori *hldE* gene displays a relatively high sequence diversity between strains, while the other genes of the cluster and their predicted protein products are highly conserved (Fig. S1B), as expected for a housekeeping function. As a basis for further functional investigations, we sought to clarify fundamental questions regarding the transcript quantities of the H. pylori heptose biosynthesis cluster genes and how those genes might be regulated. First, we determined the transcript amounts within the gene cluster for strain N6. This is the only strain where we could obtain insertion mutants in all heptose cluster genes (11). While the first genes in the cluster, presumably expressed from a housekeeping sigma^80^ promoter (38), had comparably low transcript amounts, the downstream genes in the cluster, *hldE* (HP0858) and *gmhA* (HP0857), displayed much higher transcript levels (Fig. 1B). This cannot be explained by the known transcript start sites identified in reference strain 26695 (39), where no additional start sites (primary or processed) are located directly upstream of HP0858 in the heptose gene cluster. Genome-wide transcriptome analyses revealed that the respective transcripts are strongly regulated by growth phase and by 5-methylcytosine (m^5^C) DNA methylation (40 and own unpublished data). We next addressed further internal and external influences on gene regulation and possible regulation mechanisms.

### Expression of LPS heptose biosynthesis genes and proinflammatory host cell activation are H. pylori strain specific.

Earlier work (41) established that *cag*-positive, live H. pylori strains can induce very different activation levels in gastric epithelial cells at early time points, which can now be attributed almost exclusively to heptose metabolite signaling via the ALPK1-TIFA pathway (2, 11). Hence, we asked the question of what differences in heptose biosynthesis (genetic or metabolite output) between strains might exist that can be responsible for the strain-variable activation potential.

We first tested seven different H. pylori strains from different geographical locations and bacterial populations (41) (Table 1) for their heptose gene cluster transcripts (Fig. 1C to F). Strong strain-specific differences in transcript amounts for each single gene were detected, with up to two logs of differences in absolute specific transcript quantities between strains (e.g., between N6 and Su2). The highest absolute transcript quantities in most strains, but also strongest quantitative transcript differences between strains, were obtained for transcripts of *gmhA* and *hldE* (Fig. 1C and D). In different strains, divergent patterns of relative transcript levels of heptose gene cluster genes were determined (Fig. S1C to G). According to differences in relative transcript levels of *gmhA*, *hldE*, *gmhB*, and *hldE*, strains can be grouped into two major clusters (A and B), of which, cluster A strains (such as 26695a, N6, and Su2) had the highest expression of both *gmhA* and *hldE* transcripts. In contrast, strains in cluster B (for example, L7 and Africa2, a primary *cag* pathogenicity island [*cag*PAI]-negative isolate) exhibited the highest expression of *gmhA* and *gmhB* (HP0860), whereas the second functional gene of the pathway (*hldE*) was much less expressed (Fig. 1A; Fig. S1A and C to G). This seems to suggest that the strains have evolved distinct patterns concerning enzymatic activities and potential metabolite output of the heptose pathway.

**TABLE 1:** List of strains used in the present study

Strain name	Origin	Description	Reference
H. pylori (HP) N6 wt^*a*^	hpEurope; site of isolation is France	Wild-type strain, *cag*PAI positive	56
HP N6 *cagY* (HP0527)	hpEurope	Allelic exchange insertion mutant HP0527 (*cagY*) in strain N6	11
HP N6 *hldE* (HP0858)	hpEurope	Allelic exchange insertion mutant HP0858 (*hldE*) in strain N6	11
HP N6 *hldE comp*	hpEurope	HP0858/*hldE* gene complementation in the *rdxA* locus of the HP0858 mutant in strain N6	11
HP N6 *csrA::aphA3*′*-III*	hpEurope	*csrA* (HP1442) allelic exchange insertion mutant (Materials and Methods) in N6	This study, generated according to reference 43
HP 26695a	hpEurope; site of isolation is United States	Wild-type strain, *cag*PAI positive	57
HP 26695a Δ*cag*PAI	hpEurope	*cag*PAI complete deletion by allelic exchange in strain HP 26695a	11
HP 26695a *cagY*	hpEurope	Allelic exchange insertion mutant HP0527 (*cagY*) in strain HP 26695a	This study, according to reference 11
HP L7 wt	hpAsia2, South Asia, India	Wild-type strain, *cag*PAI positive	41
HP L7 Δ*cag*PAI	hpAsia2, South Asia, India	*cag*PAI complete deletion by allelic exchange in strain L7	This study
HP J99 wt	hpAfrica1, North America	Wild-type strain, *cag*PAI positive	58, 59
HP Africa2 wt	Africa2	Wild-type strain, primary *cag*PAI negative	58
HP Su2 wt	hpNEAfrica, Northeast Africa, Sudan	Wild-type strain, *cag*PAI positive	41
HP Su2 Δ*cag*PAI	hpNEAfrica, Northeast Africa, Sudan	*cag*PAI complete deletion by allelic exchange in strain Su2	This study
HP P12 wt	hpEurope	Wild-type strain, *cag*PAI positive	60
E. coli Rosetta pLysS	Novagen	E. coli protein expression strain	Novagen

a All wild-type (wt) strains listed are minimally passaged clinical isolates.

**FIG 2:**
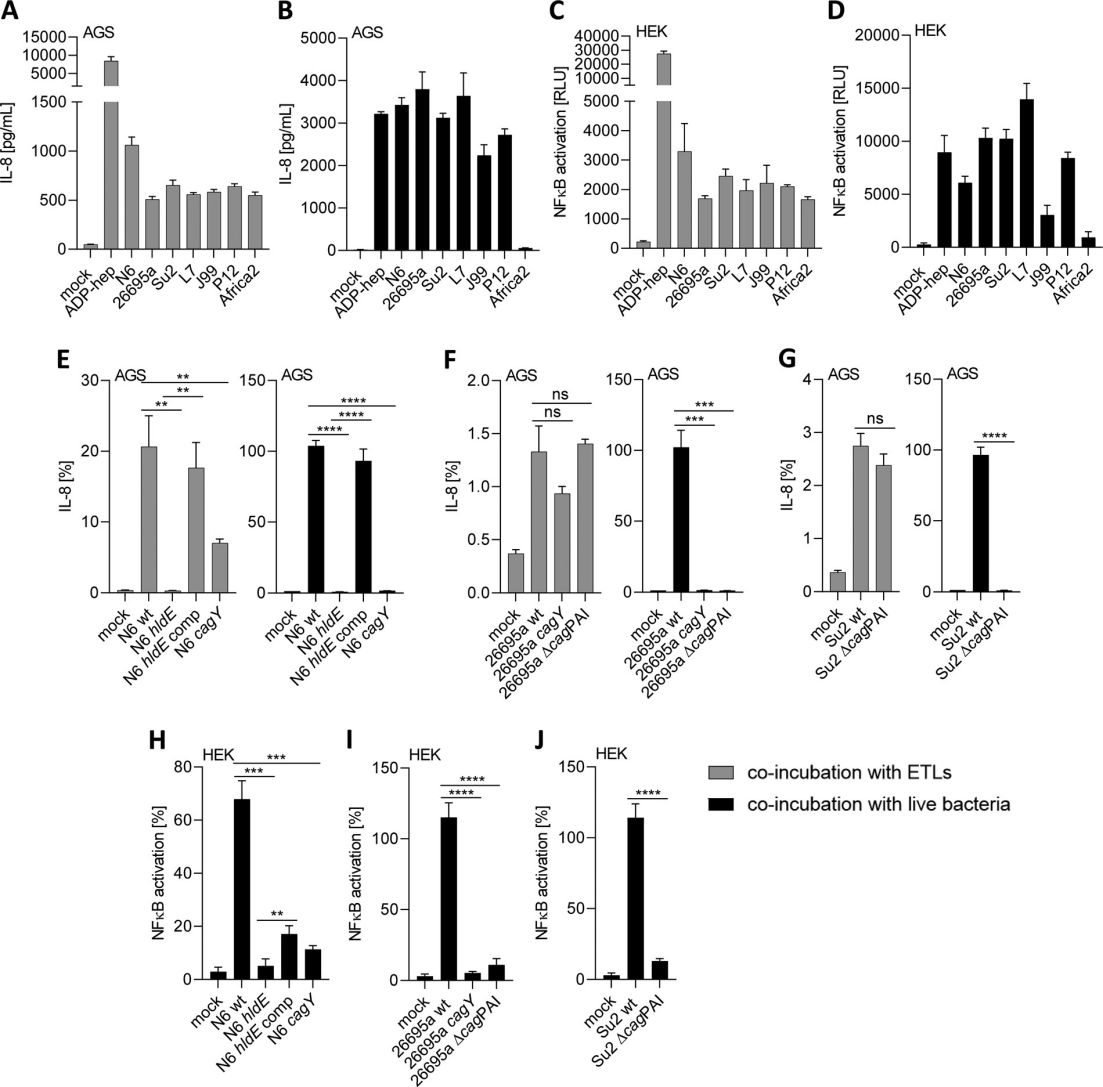
Coincubation of epithelial cells with ETLs from various H. pylori strains and corresponding live bacteria demonstrates distinct potential in proinflammatory cell activation. (A to D) Proinflammatory activation of gastric epithelial AGS cells (A and B) or HEK cells (C and D) by ETLs prepared from wild-type strains (A and C) or corresponding live bacteria (B and D) (MOI = 25). Cells were coincubated with ETLs or live bacteria for 4 h; activation was measured by IL-8 ELISA on cell culture supernatants (AGS) or luminescence measurement of the NF-κB-luc reporter cell line (HEK), respectively. Absolute values of the response are depicted for IL-8 (in pg/mL) and for luciferase measurements in relative luminescence units (RLU) as shown on the *y* axes; for comparative purposes, we applied ADP-heptose (2.5 μM) as a control stimulant for each experiment. (E to G) Activation of AGS cells after coincubation with ETLs from different H. pylori wild-type and mutant strains or the corresponding live H. pylori bacteria (MOI = 25) for 4 h. Cell activation by ETLs produced from H. pylori wild-type strains and mutants is shown in gray. AGS cell activation by live bacteria is shown in black. Cell responses were quantitated in each experiment by IL-8 ELISA from cell culture supernatants. Triplicate measurements of biological duplicate experiments are shown. Experiments were repeated at least twice with comparable results. (H to J) Proinflammatory cell activation of HEK-NF-κB_luc reporter cells after coincubation with live H. pylori bacteria for 4 h. Cell responses were quantitated by measuring luminescence. All conditions were performed in biological triplicates, and experiments were repeated at least once on different days. The results in E to J are depicted in percent values and were normalized to a ADP-heptose-exposed (at 2.5 μM, not shown) control condition performed in each experiment. Comparisons for statistically significant differences in E to J were performed between wild-type-exposed samples and each mutant-exposed sample using unpaired Student’s *t* tests. Significant *P* values are marked with asterisks; **, *P* < 0.01; ***, *P* < 0.001; ****, *P* < 0.0001; ns, not significant.

To back up the transcript analysis by other methods focusing on pathway output, we assessed proinflammatory activity of pathway products and compared them with transcript levels of the pathway genes for various strains. Enzymatically treated lysates (ETLs) of the bacteria, as demonstrated previously (11), are a good proxy measure for overall content in proinflammatory cell-active heptose metabolites in the bacteria, when grown independently of cells. In previous work, we had tested the ETLs of only two different wild-type strains (11), which appeared to have comparable proinflammatory MAMP activities, dependent on active heptose biosynthesis, but appeared to be significantly lower than for live bacteria. To clarify this preliminary result, we tested live bacteria and ETLs (treated lysates) generated from seven H. pylori wild-type strains (six *cag*PAI-positive strains and one primary *cag*PAI-negative strain) for their activity on AGS cells, MKN28 stomach epithelial cells, and HEK_luc NF-κB-luciferase reporter cells (Fig. 2; Fig. S2). Most strains’ ETLs activated proinflammatory cell responses at a comparable low level (Fig. 2A) and similar to the response to a reference amount of β-d-ADP-heptose (the readout was luciferase reporter quantification or interleukin-8 [IL-8] secretion, and the results of an ADP-heptose titration are shown in Fig. S4B). The only exception for ETL activity was strain N6, which reproducibly activated cells about 2-fold more by its cleared lysate than the other strains (Fig. 2A), suggesting a higher content in proinflammatory metabolites. Those results indicated an overall low innate activity for preformed heptose pathway products contained in bacteria grown in the absence of cells, in particular as suggested for those metabolites that can be taken up actively by the target cells (42). The live bacteria, except for the *cag*PAI-deficient strains, all activated NF-κB and elicited IL-8 secretion, with significant interstrain differences in heptose-dependent cell activation (Fig. 2B and D), as previously demonstrated (41). Proinflammatory activation by live bacteria was about 10-fold higher than ETL-mediated activation (IL-8 or NF-κB reporter quantitation) (Fig. 2). The relative proinflammatory activation patterns between the tested ETLs from different strains were comparable for the different epithelial cell lines used (Fig. 2A, 2C; Fig. S2). The results likewise highlighted that live bacteria and the active CagT4SS provide the most important means of targeted cell transport for heptose metabolites in wild-type isolates.

To assess the role of additional bacterial functions for heptose metabolite activity, we generated another set of mutants and compared them with their isogenic wild-type strains. Those included complete *cag*PAI deletion mutants (strains 26695a, Su2, and L7) (see Table 1 for strain list) and in strain N6 (where we could not obtain a *cag*PAI deletion) a *cagY* insertion mutant (defective T4SS). For N6, we also used the previously characterized isogenic *hldE* mutant and an *hldE*-complemented strain as heptose-negative and heptose-positive references, respectively (11) (Table 1). When we generated ETLs from the H. pylori isogenic mutants in the heptose pathway and from mutants deficient in T4SS assembly and tested them in comparison to the respective wild-type strain ETLs on various cell types and reporter cell lines, we found a strong difference in epithelial cell activation (Fig. 2A to G), which was entirely dependent on HldE activity and influenced by an active CagT4SS. ETLs from *cagY* mutants had an intermediate phenotype of proinflammatory cell activation on AGS cells, lower than the wild type, while ETLs from Δ*cag*PAI mutants showed no change in proinflammatory activation compared with the wild type. Live bacteria of the same strains and mutants (Fig. 2E to J) exhibited a different activation pattern compared to the ETLs. Live *hldE*-mutant bacteria showed almost no cell activation (as previously published) (11), while live bacteria from *cagY* and Δ*cag*PAI mutants (both T4SS deficient) strain independently activated cells significantly less than their corresponding parental strains. This outcome confirmed that live bacteria generally require an active T4SS for strong innate immune activation of epithelial cells. We next analyzed bacterial lysates of our set of seven diverse H. pylori strains by Western blotting (Fig. 1G) using a custom-produced polyclonal antiserum against HldE to detect strain-specific differences in protein expression. The serum was highly reactive against a protein of the predicted mass (~65 kDa) in all tested H. pylori strains but not in an *hldE* mutant (Fig. 1G). The serum identified highly variable expression of HldE protein in the different assayed H. pylori wild-type strains (Fig. 1G). We detected rather uniform HldE expression in a range of isogenic heptose (11) and *cag* mutants in H. pylori N6 (Fig. 1G).

### Regulation of heptose biosynthesis pathway transcripts in H. pylori is influenced by the absence or presence of the *cag*PAI or contact with human cells.

**FIG 3:**
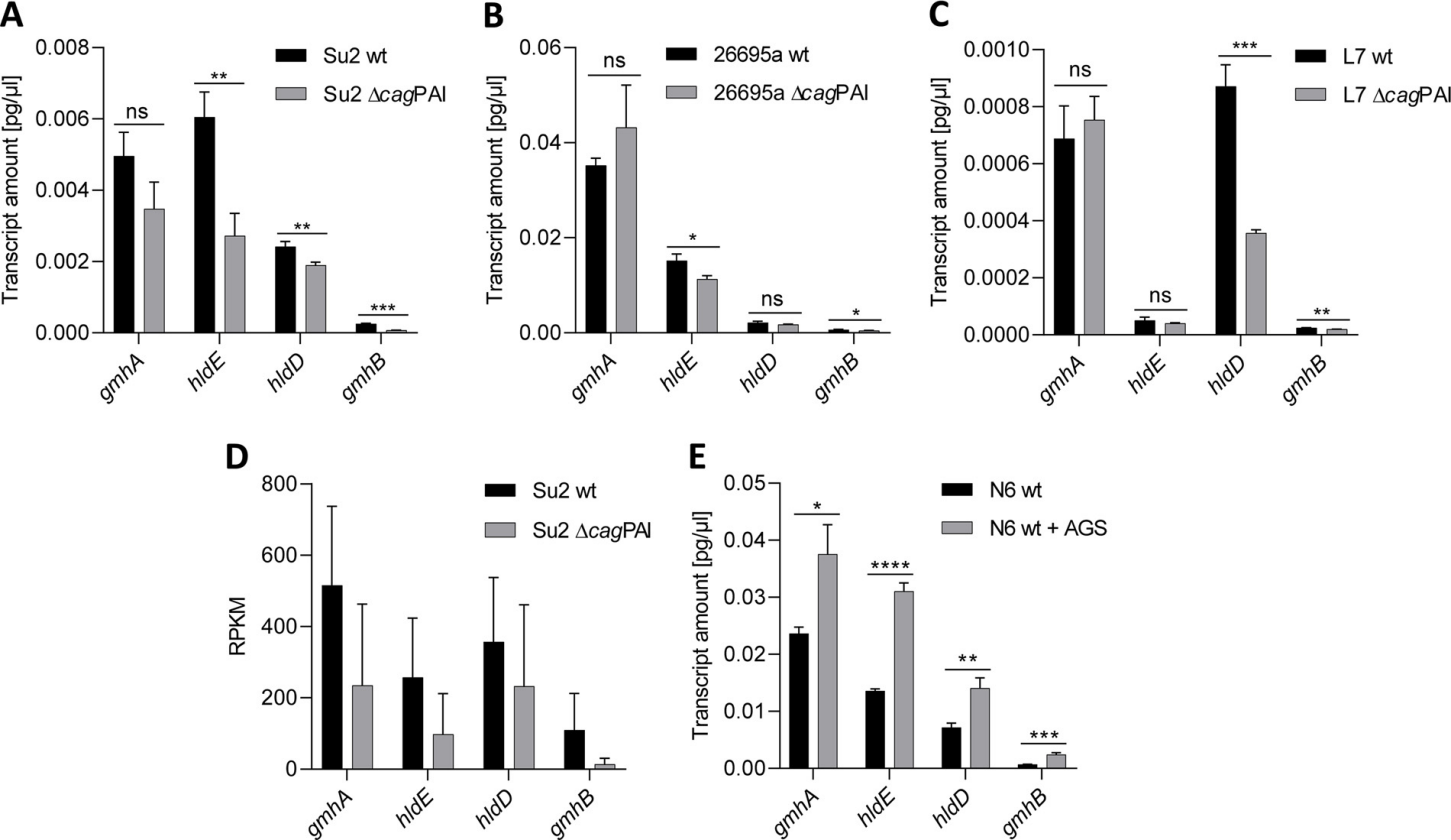
Regulation of heptose pathway transcripts in H. pylori is influenced by the presence of the *cag*PAI and contact with human gastric epithelial cells. (A) Transcript quantification (RT-qPCR) of heptose biosynthesis genes of the H. pylori Su2 wild-type strain compared with its isogenic Δ*cag*PAI mutant. (B) Transcript quantification (RT-qPCR) of heptose biosynthesis genes HP0857 (*gmhA*) through HP0860 (*gmhB*) of the H. pylori 26695a wild-type strain compared with its isogenic Δ*cag*PAI mutant. (C) Transcript quantification (RT-qPCR) of heptose biosynthesis genes of the H. pylori L7 wild-type strain compared with its isogenic Δ*cag*PAI mutant. (D) RPKM for genes *gmhA*, *hldE*, *gmhB*, and *hldD* extracted from comprehensive transcriptome data for the Su2 wild-type strain or the Su2 Δ*cag*PAI mutant. (E) Transcript amounts (RT-qPCR) for genes *gmhA*, *hldE*, *hldD*, and *gmhB* of the heptose biosynthesis cluster (Fig. S1 in the supplemental material) of the H. pylori N6 wild-type strain coincubated in the presence or absence of AGS cells (MOI = 50) in cell culture medium for 4 h. All RT-qPCR assays were performed in triplicates and normalized against 16S rRNA transcript for each sample. RT-qPCR results in A to C and E are shown as absolute values (pg/μL), using 16S rRNA amounts and the respective wild-type samples as a reference for normalization. Statistically significant differences between conditions were calculated using Student’s *t* tests. Significances are marked with asterisks; *, *P* < 0.05; **, *P* < 0.01; ***, *p* < 0.001; ****, *P* < 0.0001; ns, not significant.

We hypothesized that altered environmental conditions and the presence or functionality of the CagT4SS in H. pylori can influence the activity of the heptose biosynthesis pathway, as heptoses will be transported by the secretion system, and, possibly, the T4SS can influence production and identity of cell-active proinflammatory heptose metabolites. Therefore, we tested in a more targeted manner whether heptose gene cluster regulation and the output and activity of heptose reaction products would be modulated by mutation of the *cag*PAI (complete isogenic deletion mutants leading to CagT4SS deficiency) (Fig. 3). We found that complete deletion of the *cag*PAI had a significant reducing effect on transcript amounts, in particular on *hldE* and HP0859 (*hldD*) genes, and had a partially reducing effect on other genes of the cluster (real-time quantitative PCR [RT-qPCR]) (Fig. 3A to C). This observation was independently verified to be similar in three strains (26695a, Su2, and L7). The same pattern of downregulation of heptose biosynthesis transcripts was seen in the transcriptome data of a *cag*PAI deletion mutant compared to the parental wild-type strain (Fig. 3D).

Furthermore, using RT-qPCR, we investigated how bacteria exposed to AGS cells in the presence or absence of a functional CagT4SS regulated and expressed the LPS heptose biosynthesis gene cluster (Fig. 3E). Interestingly, we quantitated a significant upregulation of all genes of the heptose gene cluster in wild-type and *cag*PAI-deficient bacteria that were associated for 4 h with AGS cells. At 8 h of coincubation, the wild-type strain maintained a stronger upregulation (Fig. S3) than the CagT4SS-deficient mutant strain.

### Transcriptome analyses of H. pylori in the presence or absence of the *cag*PAI reveal numerous transcript changes partially associated with the carbon starvation regulator CsrA.

**FIG 4:**
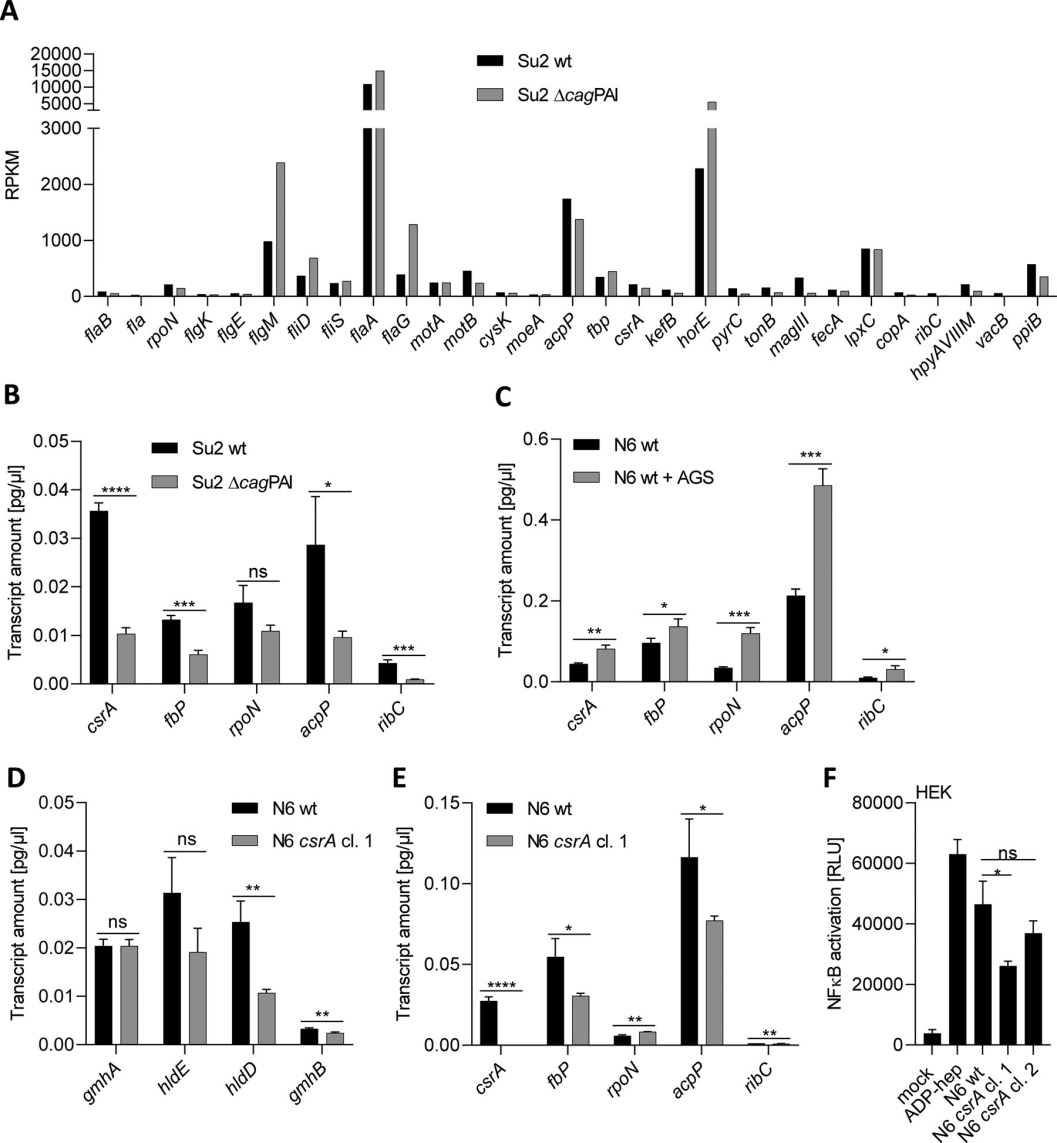
Heptose biosynthesis genes and other functional gene categories in the absence of a functional CagT4SS or in the presence of cells are regulated in association with the carbon starvation regulatory protein CsrA and in *csrA* mutants. (A) Selected RNA-seq results depicted as RPKM values from a transcriptome analysis comparing H. pylori wild type (wt), strain Su2, and its isogenic *cag*PAI deletion mutant. A representative experiment (R1) of two independently performed biological replicates is shown. The comparative data of the two replicates are fully listed in Table S2 in the supplemental material. Functionally annotated transcripts that are included in the CsrA regulon as defined in reference 43 are shown, representing functional categories of motility-related or metabolic genes. (B) The same strains cultured under the same conditions were also compared for transcript amounts of CsrA-dependent genes (*csrA*, *fbP*, *rpoN*, *acpP*, and *ribC*) by RT-qPCR. (C) H. pylori (strain N6) cultured in the presence or absence of AGS cells was also tested by RT-qPCR for selected CsrA-dependent transcripts as defined under B. (D and E) An H. pylori N6 *csrA* mutant (allelic exchange insertion) was generated, and specific gene transcripts of the heptose gene cluster and selected transcripts of the H. pylori CsrA regulon as in B and C were tested by RT-qPCR. (F) Reporter assays using HEK_NF-κB luciferase reporter cells (HEK) to detect changed proinflammatory activity of H. pylori (strain N6) *csrA* mutants on the heptose-dependent proinflammatory response. Two *csrA*-mutant clones, cl. 1 and cl. 2, were tested alongside the N6 wild-type and reference pure ADP-heptose. Statistically significant differences between conditions were tested by unpaired Student’s *t* test. Significances are marked with asterisks: *, *P* < 0.05; **, *P* < 0.01; ***, *P* < 0.001; ****, *p* < 0.0001; ns, not significant.

Bacterial transcriptome changes have the potential to provide a comprehensive perspective on gene expression. Therefore, we performed whole bacterial transcriptome analyses on H. pylori, comparing the parental strain with an isogenic *cag*PAI deletion mutant, which we hypothesized would mimic a state of intrabacterial ADP-heptose enrichment by absence of the CagT4SS. Conditions of a “closed” or nonfunctional T4SS, which we showed reduces the expression of heptose genes in three different strains (Fig. 3), can also be helpful in identifying regulators or regulons possibly involved in feedback regulation of heptose biosynthesis. The transcriptome analyses indeed revealed an influence of *cag*PAI deletion on numerous genes of different functional categories (Fig. 4A; Table 2; Table S2). Using a 1.5-fold cutoff, about 500 genes were found to be either up- or downregulated (Table S2). These included genes from different KEGG categories and also genes from the heptose biosynthesis gene cluster, with HP0858/*hldE* and HP0860/*gmhB* genes being significantly downregulated in single differential expression analyses (for information on the complete regulation data set, see Materials and Methods and Table S2). After comparison with known regulons, a number of previously reported CsrA-dependent transcripts in H. pylori (compare to the gene table in reference 43), including transcripts of motility- and metabolism-associated genes, were differentially regulated under those conditions (Fig. 4A). CsrA broadly shifts metabolism, motility, and other functions in response to environmental conditions in various bacterial species (43–46). Therefore, we also quantitated in detail the selected CsrA-related transcripts *csrA*, fructose-bisphosphatase (*fbp*), riboflavin synthase subunit (*ribF*), *rpoN* (flagellar sigma factor), and acyl carrier protein subunit P (*acpP*) by RT-qPCR comparing expression between wild-type and *cag*PAI-mutant strains and confirmed the significant regulation of most of the transcripts (Fig. 4B) between both conditions. We also compared those selected, differentially regulated, CsrA-dependent transcripts with incubation of bacteria in contact with AGS cells, which impacts the expression of heptose biosynthesis gene cluster transcripts, as shown above (Fig. 3E). Indeed, we found *acpP*, *csrA*, and the CsrA downstream genes *fbp* and *rpoN* to be significantly upregulated in bacteria coincubated with gastric epithelial cells for 4 h (Fig. 4C). Generating a *csrA* mutant, we also verified that the heptose cluster and selected CsrA regulon transcripts are indeed dependent on CsrA function, confirming their differential expression between the parental strain and the *csrA*-mutant strain by RT-qPCR (Fig. 4D and E). As a correlate of heptose biosynthesis with relation to proinflammatory cell activation, we demonstrated that *csrA* mutants show significantly lower activities than parental wild-type bacteria on NF-κB luciferase reporter cells (Fig. 4F), suggesting that not only transcript amounts but also cell-active heptose metabolite MAMP production are reduced in the mutant. Taken together, these findings demonstrated that absence of the T4SS or human cell contact exerted an inversely correlated influence on the bacteria, including regulation of heptose transcripts and other functions, in a CsrA-related manner.

**TABLE 2:** Part of comprehensive differential expression analysis for transcriptomes of H. pylori (strain Su2) in the presence or absence of the *cag*PAI

Gene name^*a*^	Max group mean	Log_2_ fold change	Fold change	*P* value
HP0172 (*moeA*)	34.88	0.79	1.73	0.04
HP0472 (*horE*)	5,524.81	1.96	3.88	0
HP0579	52.42	−2.4	−5.27	6.50E−03
HP0580	67.00	−1.39	−2.62	1.42E−03
HP0581 (*pyrC*)	141.52	−0.91	−1.89	1.01E−03
HP0601 (*flaA*)	14,938.62	1.14	2.2	0
HP0602 (*magIII*)	333.82	−1.77	−3.42	4.91E−10
HP0603	80.49	−1.86	−3.64	2.29E−03
HP0629	54.18	0.95	1.94	8.87E−05
HP0715	556.56	1.71	3.27	0
HP0751 (*flaG*)	1,284.04	2.40	5.29	0
HP0752 (*fliD*)	688.1	1.59	3.01	0
HP0753 (*fliS*)	270.58	0.87	1.82	4.53E−04
HP0754	125.88	−1.07	−2.10	0.08
HP0817	177.73	−1.08	−2.12	6.33E−03
HP0906	38.85	−1.78	−3.43	6.72E−04
HP1051	912.63	−1.88	−3.68	7.77E−16
HP1052 (*lpxC*)	848.04	0.67	1.59	1.58E−06
HP1076	71.56	-0.67	−1.59	0.18
HP1087 (*ribC*)	53.09	−1.32	−2.5	0.01
HP1122 (*flgM*)	2,389.80	1.96	3.89	0
HP1233	62.97	−1.02	−2.03	0.09
HP1248 (*vacB*)	56.09	−2.02	−4.04	1.18E−06
HP1396	164.94	1.02	2.03	4.77E−06
HP1439	13.65	−0.81	−1.75	0.61
HP1566	57.96	−3.70	−12.97	8.50E−03

a The subset of the differential expression analysis shows CsrA-dependent genes according to reference 43; only transcripts with a greater than 1.5-fold change (ratio of Su2 Δ*cag*PAI to Su2 wt [reference sample]) are included; a threshold of a maximum group mean of RPKM greater than 10 was applied. Gene name abbreviations are given in parentheses; gene numbers are those of strain 26695 (53). Complete differential expression analyses (samples B13 and B14) are contained in Table S2 in the supplemental material.

### Reconstitution of the H. pylori heptose biosynthesis pathway *in vitro* reveals various cell-active proinflammatory reaction products.

**FIG 5:**
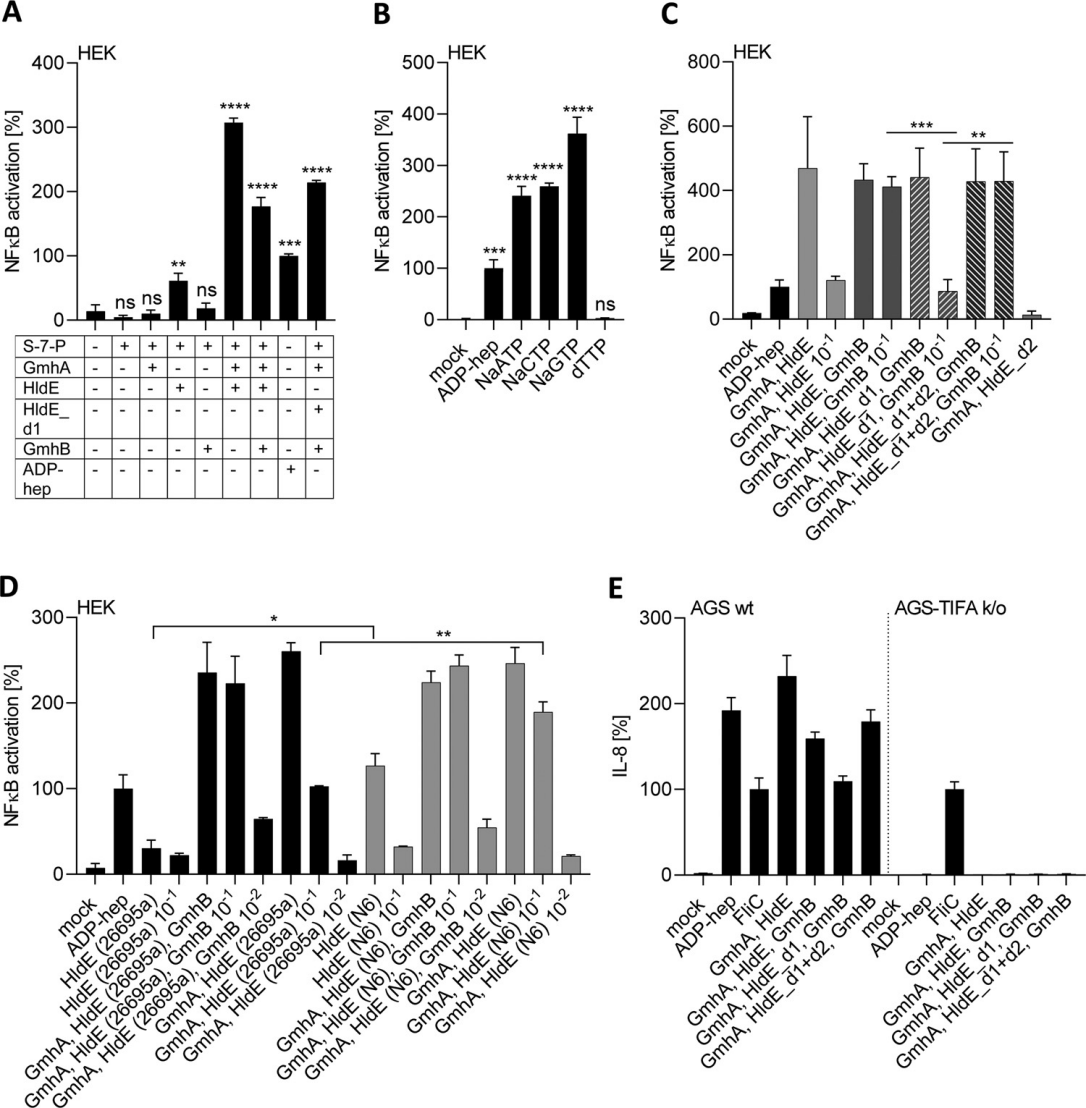
Reconstitution of H. pylori ADP-heptose biosynthetic pathway. *In vitro*-synthesized heptose metabolites of distinct enzyme combinations activate epithelial cells to different extents. (A) Proinflammatory activation of NF-κB reporter cells (HEK-NF-κB_luc) by heptose metabolites synthesized *in vitro* using recombinantly purified H. pylori proteins (strain 26695a) from substrate seduheptulose-7-phosphate (S-7-P). Absolute luciferase values are shown and are depicted in comparison to pure ADP-heptose (2.5 μM) coincubated cells (control condition). (B) HEK reporter cell NF-κB activation (normalized to 2.5 μM ADP-heptose condition, which was set to 100%) by products *in vitro* synthesized using purified enzymes GmhA, HldE, and GmhB (all from strain 26695a) and different nucleotides (all at 5.8 mM). (C) HEK_luc reporter cell NF-κB activation (normalized to the ADP-heptose control condition, set to 100%) of *in vitro* reaction products synthesized by a combination of GmhA, GmhB plus HldE (the latter as complete protein or as combined, separately purified HldE d1 and d2 domains, HldE_d1+d2), or HldE domain one (HldE_d1, from strain 26695a), or HldE domain two (HldE_d2) purified separately (nondiluted and reaction products diluted 10^−1^ are shown for each condition). (D) NF-κB activation by products synthesized *in vitro* using the three-enzyme combination with highly purified HldE (complete protein) from two different H. pylori strains, 26695a or N6, as indicated on the *y* axis (GmhA and GmhB remain in all reactions of strain 26695a). Strongly active mixtures were added nondiluted, at a dilution of 10^−1^, or at a dilution of 10^−2^. The data from the reporter cell assays in A to D were summarized from three biological replicates. (E) Proinflammatory activation of AGS wild-type (wt) and AGS-TIFA-knockout (k/o) cells by *in vitro*-synthesized heptose products, quantitated using IL-8 ELISAs on cell supernatants. Values are shown in percent and were normalized to the activation level of Salmonella FliC activation control (TLR5 ligand, 400 ng/well, set to 100%). The results shown in E were summarized from two biological replicates for each experimental condition, each quantitated in technical triplicates. Statistical evaluation of significant differences was performed using unpaired Student’s *t* tests and shows statistically significant differences. Nonsignificant differences are not indicated; *, *P* < 0.05; **, *P* < 0.01; ***, *P* < 0.001; ****, *P* < 0.0001.

The results of our regulation analyses and the observed strain differences in heptose cluster transcripts and HldE sequence and expression levels prompted us to investigate which heptose compounds and which quantities of heptose products can be synthesized by the bacteria, resulting from the different reaction steps of the heptose pathway. In addition, transfection of H. pylori ETLs into reporter cells versus the medium coincubation of reporter cells with ETLs revealed different responses, suggesting that more than one heptose product is present (see below). Using mass spectrometry (MS), Pfannkuch and colleagues had so far predominantly found β-d-ADP-heptose (with trace amounts of β-d-heptose-1,7-bisphosphate-β-d-HBP) in treated lysates of H. pylori (one strain tested) (8). ADP-heptose is a cell-permeable metabolite (4, 8). β-d-HBP was suggested to be a second cell-active heptose metabolite, possibly produced in a strain-variable manner, which is not cell permeable (8, 42). Until now, other intermediate heptose metabolites of the pathway had not been identified in H. pylori or any other bacteria. To approach the question of various products of the H. pylori enzymes, we decided to reconstitute the reaction pathway of H. pylori *in vitro* based on the three central, natively purified H. pylori enzymes of the pathway (GmhA, HldE, and GmhB) (Fig. 5; Fig. S4) from heterologous expression in E. coli. We also expressed and purified both functional domains of the bifunctional HldE enzyme (d1 and d2 domains; see Fig. S1A and Fig. S4) separately for testing. Since the H. pylori HldE enzyme, due to its clear sequence heterogeneity between strains, was the eminent candidate for pathway and product heterogeneity within the biosynthesis pathway, we also expressed and purified the full-length enzyme from two different H. pylori strains (Fig. 5). We used these proteins in various one-pot *in vitro* reactions to convert seduheptulose-7-phosphate (S7-P) substrate in the presence or absence of ATP (see Materials and Methods). As an immediate readout, we first tested the reaction products of different enzyme compositions on NF-κB luciferase reporter cells (HEK_luc epithelial cells) for determining cell-directed proinflammatory innate activation (Fig. 5A; Fig. S4). Single purified enzymes, incubated without ATP and substrate, and heat-inactivated enzymes were also tested in separate reaction mixtures as controls to exclude potential cell-activating contaminations after purification from E. coli (which all gave negative results) (Fig. S4A).

Reaction products of the successful one-pot reconstitution with three main pathway enzymes combined (GmhA, HldE, and GmhB) avidly activated cells (Fig. 5; Table 3; Fig. S4). The concentration dependency of reaction products obtained *in vitro* was also established through titration in comparison with pure β-d-ADP-heptose (Fig. S4B). We also reconstituted a reaction with only two purified enzymes, GmhA and HldE, expecting to obtain mainly β-d-HBP as a reaction product. The reaction product(s) from this second reaction unexpectedly also activated cells strongly without the need for cell transfection (Fig. 5A and C). However, titrations showed that this two-enzyme mixture was about one log less active than the three-enzyme mixture (Fig. 5C). When incubated with nucleotides other than ATP, we obtained proinflammatory product with all nucleotides except dTTP in the three-enzyme mixtures (Fig. 5B).

**TABLE 3:** Sample identifiers for NMR and mass spectrometry analyses and identification and quantification (chromatogram peak heights) of heptose metabolites detected by LC-ESI MS/MS in *in vitro* reconstitution mixtures and bacterial lysate (ETL) samples

Sample ID	Sample name (description)	Peak height ADP-heptose	RT ADP-heptose (min)^*b*^	Peak height HBP (ambiguous RT)	Peak height HMP-7/S7-P	RT HMP-7/S7-P (min)^*b*^	Peak height HMP-1	RT HMP-1 (min)^*b*^
*In vitro* reconstitution mixtures^*a*^								
P0015_S7	9 (GmhA, HldE, GmhB)	5,657,222	10.44	1,848	NP	NP	1,022,031	8.74
P0015_S8	11 (GmhA, HldE)	144,298	10.41	33,115	710,533	8.54	NP	NP
P0015_S9	9d1 (GmhA, HldE_d1, GmhB)	74,731	10.41	2,636	NP	NP	1,561,142	8.74
P0015_S10	11d1 (GmhA, HldE_d1)	12,537	10.40	48,309	836,843	8.54	NP	NP
P0015_S11	11N6 (GmhA, N6_HldE)	346,331	10.39	4,251	1,546,445	8.54	NP	NP
P0015_S12	GmhA only	5,476	10.41	1,723	1,884,683	8.55	NP	NP
P0015_S13	HldE only	1,641	10.40	1,181	2,136,561	8.54	NP	NP
							**Peak height HMPv**	
H. pylori lysates (ETLs)								
P0015_S14	N6 wt 1 (replicate 1)	14,189	10.42	1,239.96	837	8.70	421.46	8.80762
P0015_S15	N6 *gmhA* (11)	318	10.47	ND	768	8.68	ND	ND
P0015_S16	N6 *hldE* (11)	192	10.40	ND	649	8.68	ND	ND
P0015_S17	N6 *hldE* comp (11)	7,858	10.42	ND	650	8.67	340.96	8.80762
P0015_S18	N6 *gmhB* (11)	217	10.40	ND	697	8.69	ND	ND
P0015_S19	26695a wt 1 (replicate 1) (11)	7,943	10.41	ND	474	8.65	399.94	8.80762
P0015_S40	N6 wt 2 (replicate 2)	15,091	10.29	—	—	—	—	—
P0015_S42	26695a wt 2 (replicate 2) (11)	5,926	10.29	—	—	—	—	—
P0015_S45	Su2 wt	8,928	10.29	—	—	—	—	—
P0015_S47	L7 wt	3,600	10.29	—	—	—	—	—
P0015_S49	Africa2	3,137	10.28	—	—	—	—	—
P0015_S50	J99 wt	5,385	10.30	—	—	—	—	—
P0015_S51	P12 wt	31,530	10.29	—	—	—	—	—
Reference compounds								
Ref_ADP-Heptose (1 μM)		17,208	10.40	NP	NP	NP	NP	NP
S7-P (2.5 μM) pHILIC		NP	NP	NP	49,455	8.56	NP	NP

a *In vitro* reconstitution mixtures (Materials and Methods) contain purified enzymes from H. pylori strain 26695a if not indicated otherwise.

b RT, retention time; NP, not present; ND, clear peak not detectable; —, not measured.

For HldE enzymes purified recombinantly from two different strains, which show considerable amino acid sequence diversity (Fig. S1B), both produced cell-active proinflammatory compounds. However, the enzymatic action of HldE from N6 resulted in a significantly stronger cell activity in the two-enzyme reaction than HldE from strain 26695 (Fig. 5D). Even for HldE-only reaction mixtures, we were able to produce low-level cell-active output (Fig. 5D). In this case, the resulting activity was almost zero for 26695 HldE and was significantly higher for HldE from N6 strain (Fig. 5D). For all reaction mixtures, we verified whether the resulting activity was dependent on the ALPK1-TIFA pathway by comparing their activity on AGS wild-type and AGS TIFA-knockout (k/o) cells (11) using IL-8 release (enzyme-linked immunosorbent assay [ELISA]) as a readout. The TIFA-k/o cells showed no proinflammatory activation by our mixtures, confirming lack of product activity in the absence of TIFA-mediated signaling, while an alternative innate NF-κB stimulant, Salmonella FliC flagellin (TLR5 ligand), was positive (Fig. 5E).

### Proinflammatory heptose pathway products identified as β-d-ADP-heptose and β-HMP-1 using NMR and mass spectrometry.

**FIG 6:**
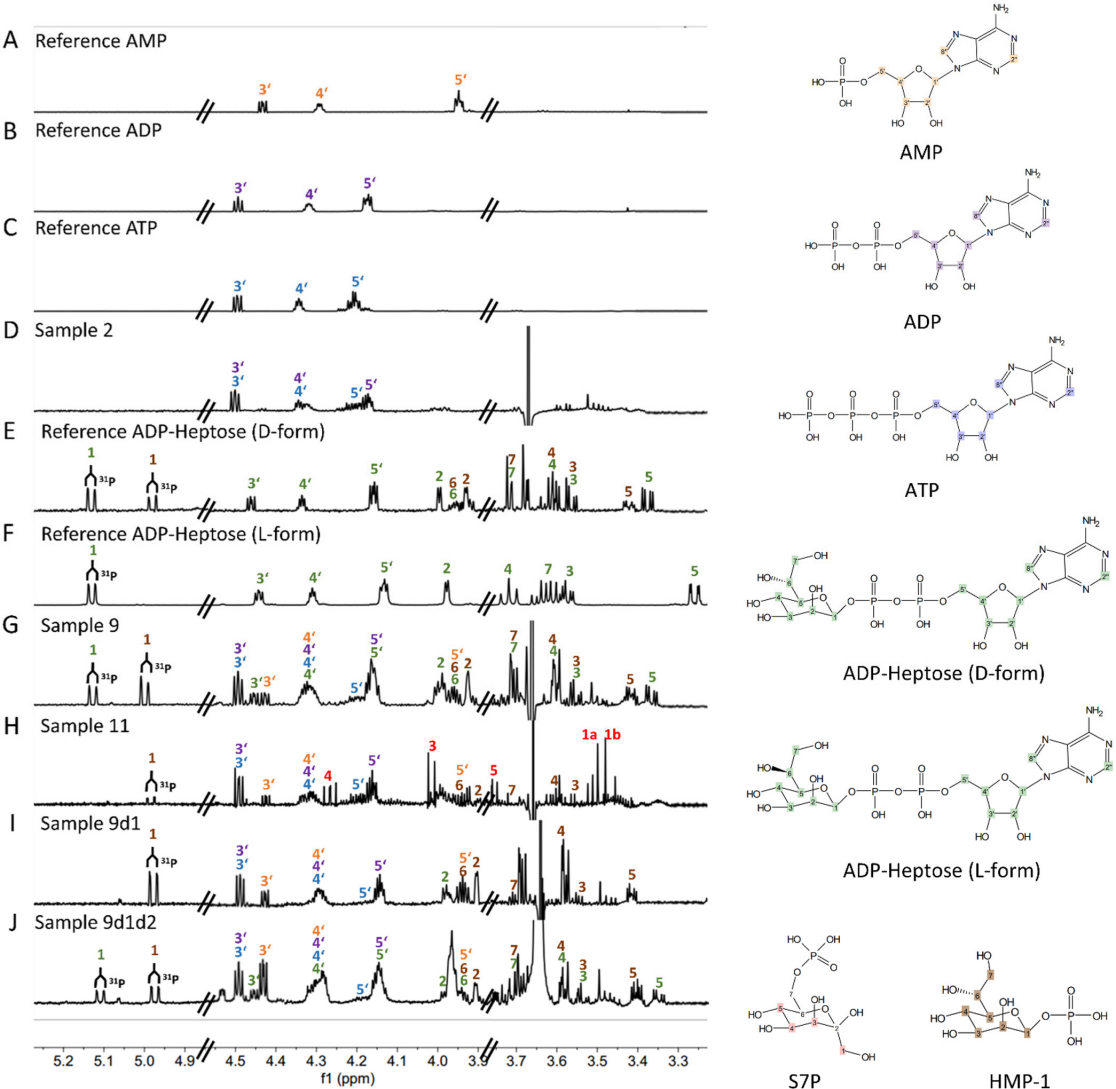
NMR analysis of reaction products of the *in vitro* reconstituted H. pylori ADP-heptose biosynthesis pathway identifies the new cell-active product β-HMP-1. *In vitro* reconstitution of the H. pylori ADP-heptose biosynthetic pathway was performed in one-pot reaction mixtures with various enzyme combinations as described in Fig. 5, Fig. S4 in the supplemental material, Table 3 (sample identities), and the Materials and Methods. NMR was performed as described fully in the Materials and Methods. (A to F) Control conditions and control compounds as indicated in the upper left corners analyzed in pure form (ATP, AMP, ADP, β-d-ADP-heptose, and β-l-ADP-heptose; sample 2 [D] is a control mixture without any substrate). The nomenclature of one-pot reactions (D and G to J) was as follows: sample 2, control sample with ATP and three enzymes but no substrate; sample 9, three enzymes, GmhA, HldE, GmhB, ATP, and sedoheptulose-7-P (substrate); sample 11, two enzymes, GmhA, HldE, ATP, and substrate; sample 9d1, three enzymes with HldE d1 only, ATP, and substrate; sample 9d1d2, three enzymes with HldE d1 and d2 (purified and added separately), ATP, and substrate. All enzymes used here were cloned from strain 26695a. Structures of compounds and reaction products and their respective atoms are indicated on the right; atoms are numbered and labeled in different colors. Color-coded numbered peaks in the histograms correspond to the respective atoms of the input and output compounds, shown on the right, that were detected. Detailed results for references and samples are listed in Table S3 in the supplemental material.

To assign compound structures to the one-pot reaction products, we then used ^1^H NMR analysis of the reaction mixtures containing different sets of recombinant enzymes from H. pylori (Fig. 6). Based on the specific chemical shifts, coupling constants, and scalar correlations observed in two-dimensional correlation spectroscopy (COSY) experiments (Table S3), β-d-ADP-heptose was identified as the main reaction product of the combined action of GmhA, HldE, and GmhB proteins when ATP was supplied as the energizing nucleotide (Fig. 6G). Comparing with reference compounds (β-d-ADP-heptose and β-l-ADP-heptose; confirmed by NMR) (Fig. 6E and F), we showed that the ADP-heptose product of the H. pylori enzyme reconstruction reaction was of the d-form (ADP-d-glycero-β-d-manno-heptose) (Fig. 6G). In the two-enzyme reaction (GmhA and HldE), NMR spectrometry identified another, unexpected, cell-activating product, which was not identical to β-d-HBP but was identified as predominantly β-heptose-monophosphate-1 (β-HMP-1), in addition to the reaction mixture ingredients (Fig. 6H). Dilution titration of the two-enzyme reaction mixture containing β-HMP-1 yielded about 10-fold lower activity on cells (without transfection) than the three-enzyme reconstitution (Fig. S4B). Since previous studies had suggested that β-d-HBP is preferentially produced by HldE, we supplemented the HldE d1 and d2 domains (Fig. S1A and S4) separately in the reconstitution. The d2 domain alone in combination with GmhA only (Fig. 5C) or with both GmhA and GmhB (not shown) did not yield any active product. Using solely the d1 domain of the HldE enzyme in combination with GmhA, we obtained a proinflammatory product that we also identified as β-HMP-1 by NMR and no detectable β-d-HBP, suggesting that the HldE d1 domain activity, together with GmhA, was sufficient for HMP-1 production (Fig. 5C and 6I). The d1 and d2 domains combined behaved similar to the complete HldE enzyme in all reaction mixtures, yielding mainly ADP-heptose and a residual amount of β-HMP-1 but no detectable β-d-HBP (Fig. 6J). The alternative nucleotides Na-GTP and Na-CTP, supplemented instead of ATP in the three-enzyme mixtures, did not yield detectable amounts of nucleotidyl-heptose despite pronounced proinflammatory cell activity; instead, β-HMP-1 was also revealed by NMR (not shown).

**FIG 7:**
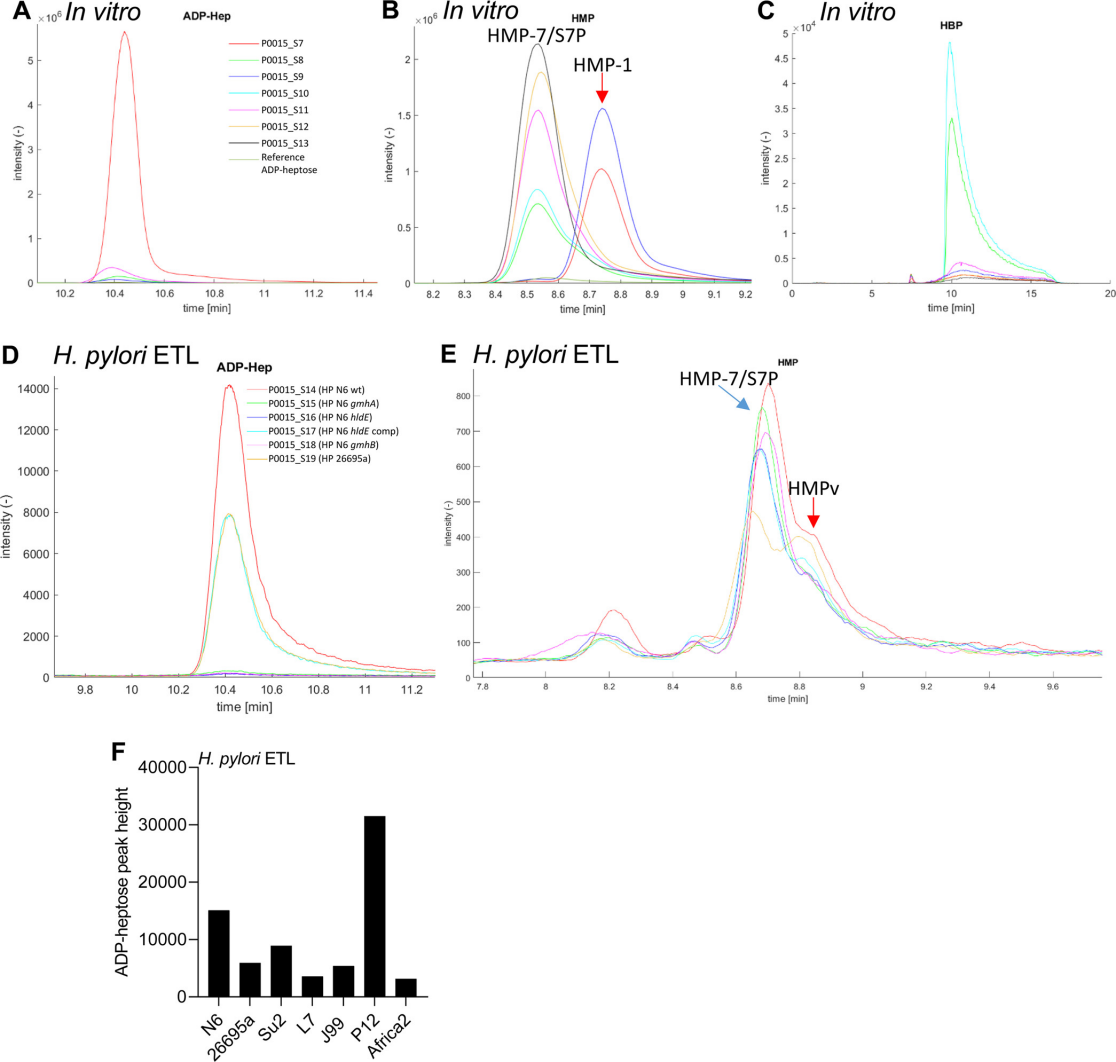
Mass spectrometry analysis (LC-ESI-MS-MS) confirms reaction products of the *in vitro*-reconstituted H. pylori ADP-heptose biosynthesis pathway and identify a new proinflammatory product β-HMP-1 and strain-specific quantities of ADP-heptose and other heptose metabolites in H. pylori lysates. *In vitro* reconstitution of the H. pylori ADP-heptose biosynthetic pathway was performed in one-pot reaction mixtures with various enzyme combinations (enzymes cloned from 26695a) as described in Fig. 5, Fig. S4 in the supplemental material, Table 3, and the Materials and Methods. Mass spectrometry was performed as described in the Materials and Methods. (A to C) Detection and quantitation of ADP-heptose (A), HMP variants (B), and HBP (C) in various one-pot mixtures of purified H. pylori enzymes (see Table 3). (D and E) show the detection and quantitation of ADP-heptose and HMP variants by mass spectrometry in H. pylori lysates (ETLs). Same color codes of chromatogram curves correspond to the same samples in A, B, and C, respectively, and in D and E, respectively. In D, samples S14 (N6 wt ETL), S17 (N6 *hldE* comp ETL) and S19 (26695a wt ETL) show strain-specific high amounts of ADP-heptose. In E, the same samples also show an increased shoulder peak compared to the other samples at the approximate retention time of heptose-monophosphate-1 (HMP-1). This shoulder is designated here as a heptose monophosphate variant, not identical to HMP-7 (HMPv), since the assignment of the shoulder peak was ambiguous. Peaks in the chromatograms are labeled with the respective compound designations (input and output compounds). See Table 3 for full descriptions of samples, compound identifications, and quantifications (peak height). (F) shows diverse ADP-heptose content (peak heights) in ETLs of seven different H. pylori wild type strains, quantitated by mass spectrometry.

Due to the intrinsic low sensitivity of NMR spectroscopy in conjunction with severe signal overlap in the NMR spectra of crude extraction mixtures, we were not able to detect any heptose compounds in H. pylori lysates by NMR. We therefore turned to mass spectrometry (liquid chromatography-electrospray ionization tandem mass spectrometry [LC-ESI MS/MS]) to test both *in vitro* reconstitution mixtures and H. pylori ETLs (see sample overview in Table 3) to verify compound structures and refine their detection and relative quantitation. In the *in vitro* reconstitution samples, depending on their composition, we were able to clearly detect and quantitate ADP-heptose (Fig. 7A), HBP (Fig. 7C), and the heptose-monophosphates (Fig. 7B). In these samples, the two groups of heptose-monophosphates (HMP-7/S7-P and HMP-1, respectively) can be clearly distinguished from each other by different retention times (Fig. 7B; Table 3). The largest amount of ADP-heptose product was quantitated in the three-enzyme mixture containing full-length HldE. Most HMP-1 was detected and quantitated in the three-enzyme mixtures containing either full-length HldE or HldE d1 domain only (Fig. 7B; Table 3). HBP was detected predominantly in the two-enzyme mixtures containing GmhA and HldE or GmhA and HldE d1 (Fig. 7C; Table 3).

In H. pylori ETLs, mass spectrometry also detected a distinct heptose monophosphate species (HMPv, probably β-HMP-1) (Fig. 7E) as a novel compound (shoulder peak in the chromatogram, matching the retention time in the reconstitution mixture containing β-HMP-1) in addition to ADP-heptose (Fig. 7D), both in the H. pylori wild-type strain and in ETLs of an HldE overexpression strain (Fig. 7E; Table 3). Interestingly, greatly divergent amounts of specific metabolites in two different wild-type isolates were quantitated by LC-MS, with respect to amounts of ADP-heptose and the probable HMP-1 monophosphate species (Table 3). Differences in ADP-heptose quantities between N6 and 26695a strain lysates were more than 20-fold (peak heights are in Table 3), and ADP-heptose content in lysates also varied quantitatively for a broader range of diverse wild-type clinical isolates (Fig. 7F). Similarly, proposed HMP-1 heptose-monophosphate showed quantitative differences between the two strains (Table 3). This clearly corresponded with a significant difference in proinflammatory cell activity between both strains (live and lysates) (Fig. 2). HBP, while clearly revealed in enzymatic reconstitution mixtures (Table 3), was poorly detectable by LC-MS in bacterial lysates, probably due to low concentrations and compound instability.

### Medium supplementation versus cell transfection of reaction products of *in vitro* reconstitution and H. pylori ETLs activate cells differently: further evidence for differentially active heptose products.

**FIG 8:**
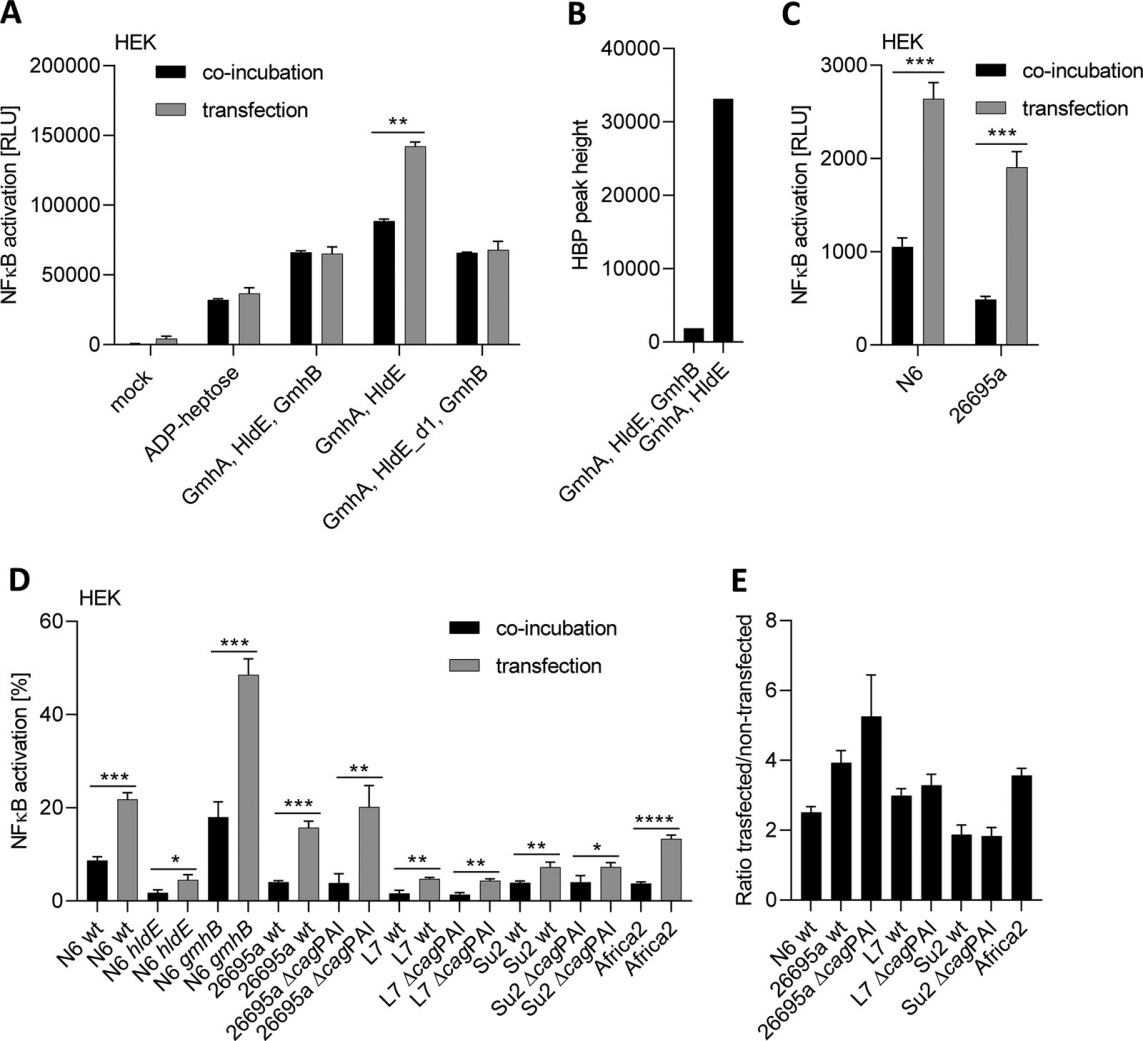
Transfection improves the proinflammatory activity of partially reconstituted heptose reaction (GmhA and HldE) and of some bacterial ETLs. (A) Activation of HEK-NF-κB_luc reporter cells after coincubation or transfection by reconstituted heptose mixtures produced using the recombinant H. pylori enzymes GmhA, HldE (complete enzyme or separate d1), and GmhB in different combinations (4 h of cell coincubation). (B) Comparative quantitation of HBP by LC-MS/MS in the reconstitution mixtures of three heptose pathway enzymes versus two enzymes (correspond to samples S7 and S8 in Table 3). The three-enzyme mixture contains very little HBP, while the two-enzyme mixture shows a more than 10-fold increase in HBP. (C) Activation potential of H. pylori ETLs on HEK-NF-κB_luc reporter cells after coincubation or transfection of enzymatically treated lysates (ETLs) of the H. pylori wild-type strains N6 and 26695a (4 h of coincubation). Reporter activation is quantitated in absolute luminescence values (RLU) in A and C. (D) Differences in proinflammatory cell activation (HEK-NF-κB_luc) between coincubation with and transfection (T) of various H. pylori strains’ ETLs. Strain and mutant designations (wt indicates wild type of strains N6, 26695a, L7, SU2, and Africa2; mutants are designated by respective gene names) conform to previous figures. Incubation time was 4 h for all conditions; all values (shown in percent) were normalized to ADP-heptose coincubation of the same experiment (2.5 μM, not shown), which was set to 100%. Experiments to detect NF-κB activation in A, C, and D were performed in two (A) or three biological replicates each and were repeated at least once on different days. Statistically significant differences between conditions of coincubation and transfection were calculated using Student’s *t* tests. Significances are marked with asterisks; *, *P* < 0.05; **, *P* < 0.01; ***, *P* < 0.001; ****, *P* < 0.0001. (E) Calculation of ratios between coincubated and transfected conditions for response to selected strains’ ETLs (only wild-type strains and isogenic T4SS-deficient mutants, same coincubation conditions as in D).

When we coincubated reporter cells with reaction products from the *in vitro* reconstitution reactions, we observed strong proinflammatory cell activation using the three-enzyme reconstitution (described above), mostly producing β-d-ADP-heptose according to NMR results. This activity was absent when TIFA-k/o cells were used, which cannot mount an innate immune response toward heptose metabolites (Fig. 5E), clearly assigning the activity to the heptose product(s). The cell-directed proinflammatory activity was markedly lower when coincubating reporter cells with reaction products from the two-enzyme reaction (enriched in β-HMP-1 and β-d-HBP) by merely supplementing the medium. To gather more evidence for a mixture of active products in some of the reactions, we transfected pathway reconstitution products and bacterial ETLs into the HEK-NF-κB_luc reporter cell line (Fig. 8A). While transfection of the three-enzyme pathway product (containing mostly ADP-heptose as per our biochemical analyses) elicited about the same amplitude of response as coincubating the same amount in the medium, the products of the two-enzyme reconstitution behaved differently; in this case, the transfection of the product elicited at least 3-fold higher activity in the reporter cells than medium supplementation with the same reaction products (Fig. 8A). When analyzed by mass spectrometry, the two-enzyme reaction contained markedly higher amounts of the nonpermeable metabolite β-d-HBP than the three-enzyme mixture (Fig. 8B; Table 3). We also compared the proinflammatory activity of bacterial ETLs collected from five different H. pylori strains (26695a, Su2, N6, L7, and Africa2) and isogenic Δ*cag*PAI or *cagY* mutants of three strains (Fig. 8C and D) on cells with and without lipofectamine transfection. In those experiments, the ETLs always activated significantly more when transfected (Fig. 8C and D). Strain differences were determined for the same amount of ETLs using the ratio between transfected and nontransfected, quantitating how strongly the activation was increased after transfection. The ratios ranged from slightly above or below a 2-fold increase for N6 and Su2 (Fig. 8E) to between 3-fold and 4-fold for 26695a, L7, and Africa2. This corresponds to our analyses of ETLs containing (i) cell-permeable ADP-heptose (shown before in reference 8), (ii) a proinflammatory active monophosphate compound (HMPv), likely HMP-1, which also has good cell permeation activity (42), and (iii) at least one other heptose metabolite, which is less cell permeable (suggested to be HBP) (5, 8). *In vitro* one-pot reactions that cannot produce β-d-HBP (e.g., a mixture including GmhA, the d1 domain of HldE, and GmhB) and that contained HMP-1 (by NMR and LC-MS) as the only active product did not result in increased activation after cell transfection. We also tested several isogenic heptose biosynthesis and Δ*cag*PAI-mutant lysates in comparison with their wild-type parent ETLs by cell transfection (three strains). Most mutants did not behave differently from their wild-type parent (Fig. 8D and E). For strain N6, the isogenic *gmhB*-mutant ETL (expected to increase HBP product) displayed a substantial activity increase by transfection over nontransfected conditions (Fig. 8D) and a net increase of activity over the parent strain. Interestingly, testing the 26695a *cag*PAI deletion mutant ETL revealed a stronger NF-κB activity increase by transfection over no transfection than the 26695a wild-type ETL (Fig. 8E). *cag*PAI deletion would mimic a closed T4SS. These results strongly suggest that the presence of the *cag*PAI can affect the production of metabolites that are less permeable than ADP-heptose.

## DISCUSSION

Heptose metabolites from diverse bacteria have recently been characterized as MAMPs/pathogen-associated molecular patterns with strong proinflammatory activating potential for human myeloid and epithelial cells (12, 47). Some heptose metabolites, such as ADP-heptose, can be actively taken up as solutes after they have been released into the environment (5, 7, 8, 42). A number of pathogenic bacteria, including H. pylori and some *Enterobacteriaceae*, do not release a substantial amount of heptose metabolites into the medium but use their host-directed secretion systems (T4SS and T3SS) for direct transfer of the metabolites into host cells (4, 8, 11, 12, 14). This mode of targeted metabolite transport raises the obvious questions of whether and how bacteria use active mechanisms to regulate the activating metabolite biosynthesis, possibly in a strain- and cell contact-specific manner. Envisaged mechanisms would comprise specific sensing of environmental cues and subsequently lead to increased activation of heptose biosynthesis genes after host cell contact.

Using the model organism H. pylori, which translocates ADP-heptose and possibly other heptose metabolites via its type 4 secretion system, we have addressed several questions regarding strain diversity in heptose gene regulation, heptose metabolite generation, and variable proinflammatory effects of bacterial heptoses on host cells. First, we obtained a fundamental knowledge on strain diversity in transcript amounts and transcriptional and posttranscriptional regulation mechanisms of the H. pylori heptose biosynthesis gene cluster. Transcript amounts varied between strains and between the genes of the cluster in each tested strain, with the last genes of the cluster, and the most relevant genes for generating proinflammatory heptose intermediate products (11), *gmhA* and/or *hldE*, frequently possessing the highest transcript activities. Both the absolute transcript amounts of each gene and relative expression patterns of the cluster genes were extraordinarily strain variable. In particular, we found strains with very high transcript amounts specifically of *gmhA* and *hldE*, while other strains, such as L7 (Asian origin) and Africa2 (a primary *cag*PAI-negative strain), had higher transcript amounts selectively of both *gmhA* and *gmhB* but not of *hldE*. These observed expression differences can explain some of the strain differences in proinflammatory bacterial lysate activity and in cell responses induced by live bacteria that have been observed in this work and before (41). In addition, while *gmhA*, *gmhB*, and *hldD* did not differ markedly in sequence between H. pylori strains, the bifunctional *hldE* gene, crucial for the generation of proinflammatory cell-active heptose metabolites (3, 8, 10, 11), showed a comparably strong interstrain DNA and derived amino acid sequence variability. Interestingly, using a specific antiserum against HldE, we could also pin down marked strain-specific differences in HldE protein content. The finding that the different strains had a very diverse heptose-dependent proinflammatory activation potential on epithelial cells as shown here and previously (11, 41) is an expected outcome of expression differences and HldE sequence diversity. HldE protein expression in different mutants (*cagY* and Δ*cag*PAI) of the same strain appeared not much changed except for the HldE mutant. Taken together, our findings of transcript profile diversity and strain-variable HldE sequence and protein content suggest a major role of this bifunctional enzyme in host interaction. Since bacterial lysates do not permit the detection of metabolite transport differences of live bacteria, we still have less insight into active metabolite production and content in live bacteria in the presence of target cells. Upregulation of the heptose cluster genes in wild-type bacteria with an active T4SS in the presence of cells is highly suggestive of increased heptose metabolite biosynthesis after cell contact.

To identify novel proinflammatory heptose metabolites that H. pylori can variably produce, and to identify strain-specific differences in enzyme activities as one possible explanation for interstrain variation of active metabolite content, we reconstituted the heptose biosynthesis pathway in one-pot reaction mixtures. We used purified HldE enzyme from two different H. pylori strains (26685 or N6) and two separate HldE domains. When we tested the metabolic reaction products on NF-κB reporter cells, the fully reconstituted pathway mixture (the three enzymes GmhA, HldE, and GmhB combined) as well as the combination of GmhA and HldE (two enzymes only) both produced proinflammatory cell-permeable active metabolites, although the latter reaction cannot form ADP-heptose. Transfection of reporter cells with pathway reconstitution products revealed that the two-enzyme reaction products acted much stronger after transfection, while the three-enzyme product (predominantly β-d-ADP-heptose and HMP-1 according to NMR and mass spectrometry analyses) showed no difference between transfection and supplementation in the cell medium. The H. pylori pathway had been only partially enzymatically reconstituted *in vitro* before the present study, using synthetic β-HMP-7 or β-HMP-1 as substrates and GmhB and/or HldE as purified enzymes (8). In the latter study, only β-d-ADP-heptose was identified as the reaction end product in H. pylori lysates (using mass spectrometry), which is cell permeable, while β-d-HBP, an intermediate pathway product, is not (5). The reconstituted heptose pathway from Campylobacter jejuni, in a three-enzyme mixture, was previously assessed to activate cells by external supplementation (7), while the latter study did not characterize the reaction products further. We established differences in the processive enzyme activities of HldE proteins from two different strains (26695a and N6), which show considerable amino acid sequence diversity, using the one-pot mixtures followed by LC-MS analysis of the products. HldE of strain N6, alternatively added to the enzyme mixtures, was significantly more active, even as a single enzyme or in the two-enzyme combination with GmhA and produced significantly more ADP-heptose detectable by mass spectrometry, both in the enzyme mixtures and in H. pylori lysates (Fig. 7D). The findings of interstrain transcript, regulatory, and enzyme variation in the heptose biosynthesis pathway points to a variety of factors that can influence strain diversity in cell-directed metabolites and proinflammatory activity.

In the present study, we could now also unequivocally assign major reaction products formed in one-pot reaction mixtures containing recombinant H. pylori enzymes, and in H. pylori lysates, using both NMR spectroscopy and mass spectrometry. In the two-enzyme mixtures, we identified β-d-HBP by mass spectrometry. By NMR, this reaction mixture also identified a second proinflammatory metabolite heptose-monophosphate variant β-HMP-1, which had been synthesized *in vitro* as a novel cell-active heptose product previously (42) but had not yet been identified in bacteria directly. β-HMP-1 can theoretically be formed already in the second enzymatic step of the biosynthesis reaction, which we could confirm by NMR in a mixture using only the HP0858 d1 domain in addition to GmhA. By mass spectrometry, HMP-1 was not identified in the two-enzyme mixtures, which is probably due to detection limits inherent in the methodology. Both by NMR and mass spectrometry, HMP-1 and β-d-ADP-heptose were readily detectable in the three-enzyme mixtures. In H. pylori lysates, depending on strain and mutant identity, we quantitated as main cell-active products both ADP-heptose and a probable monophosphate (likely HMP-1), which has never been identified directly in bacteria. Particularly intriguing were the strong differences in amounts of ADP-heptose and proinflammatory heptose-monophosphate quantities between tested wild-type strains. From transfection experiments of lysates and the two-enzyme preparations, we assume that, additionally, a third active compound other than ADP-heptose and probable HMP-1 is contained. HBP, which was only detectable in one tested strain (N6) by mass spectrometry, has been detected before in low quantities and was found to not be cell permeable (4, 8, 42). We established that the activities and metabolite content of wild-type lysates and *in vitro* reaction mixtures containing HldE proteins from two different H. pylori isolates (N6 and 26695), which show considerable amino acid sequence diversity, were different. HldE reaction mixtures of strain N6 were significantly more enzymatically active, even as a single enzyme or in the two-enzyme combination with GmhA, to produce HMP-1 or ADP-heptose. This matched to the higher detectable content of metabolites in strain N6 bacterial lysates, which was only exceeded by another frequently used strain P12 (8). Strain differences in the production of β-d-ADP-heptose and other heptose metabolites by H. pylori can therefore be detected and partially quantitated directly in lysates. Strain differences in heptose metabolite production can also be inferred in our present study from (i) distinct transcript amounts of various heptose cluster genes between different strains, (ii) varying enzyme activities, or (iii) the strain-variable ratios between lysate-transfected and coincubated conditions. In any case, for the production of cell-active intermediate pathway metabolites, the central bifunctional enzyme HldE is essential. Through our *in vitro* reconstitution, we also collected evidence that domain d1 of HldE might, strain-specifically (e.g., in N6), act not only as a kinase but also as a phosphatase *in vitro*. This finding may resolve the conundrum that in distinct live H. pylori isolates, the phenotype of heptose metabolite-dependent cell activation of HP0860 (*gmhB*) mutants was found to be strain-dependently distinct (8, 11). Since we demonstrated that the H. pylori heptose biosynthesis pathway can produce a proinflammatory intermediate metabolite HMP-1 in the first two steps already with only GmhA and HldE, the next enzymatic steps may, in principle, not be necessary to produce proinflammatory metabolites.

Expressed separately, the two domains of the HldE enzyme, corresponding to the different enzymatic domains, had differential effects when used as part of the reconstitution reaction; single d2 addition in the three-enzyme mixture did not lead to any cell-active product, while single d1 addition also produced detectable cell-active β-HMP-1 (NMR and mass spectrometry). Activity of the latter reaction product was not higher when transfected into cells, different from the products of the GmhA and HldE two-enzyme reconstitution, supporting again the notion that a nonpermeable product (HBP) causes the additional activation. The use of different nucleotides as cofactors in the reaction revealed that nucleotides are essential cofactors for all steps of the reactions to proceed. Without nucleotides, no products are revealed. Second, nucleotides other than ATP (CTP and GTP) can serve as cofactors of the enzymes in the first two reaction steps as efficiently as ATP but cannot be transferred into nucleotidyl-heptose by HldE, as the reaction product then stopped at β-HMP-1. Adekoya et al. (42) demonstrated that, when provided at equal molarities with transfection, β-HMP-1 was about equally active on HEK epithelial cells as β-d-HBP, while HMP-7, the primary intermediate reaction product generated from S7-P by GmhA (also detected in some of our samples), was not cell active at all. Our coincubation results also confirmed that various human cell types can take up ADP-heptose and β-HMP-1 metabolites from the medium without the need of a bacterial transport system (12).

In addition to strain-specific traits in transcript amounts and enzyme and lysate activities of the heptose biosynthesis gene cluster, we identified a role of the presence and activity of the *cag*PAI and the CagT4SS. In mutants not possessing the *cag*PAI, the heptose cluster genes were expressed less than in the corresponding wild-type strains. A closed or absent secretion system, inducing feedback regulation, broadly influenced bacterial gene regulation in different functional categories, emphasizing metabolic and motility functions. The presence of cells also modulated bacterial gene regulation and, in turn, upregulated heptose pathway transcripts. Our regulation analyses therefore suggest that heptose cluster transcript activities and pathway output can be adjusted according to T4SS activity and the presence of cells. Prompted by the pathway analysis of the comprehensive differential transcriptomes in *cag*PAI-deleted bacteria, we obtained evidence that the modulation of the heptose gene cluster in H. pylori is at least partially under CsrA regulation (43, 48–50). CsrA is an important metabolic and global posttranscriptional bacterial regulator acting on RNA, which broadly influences metabolic pathways and motility functions in various bacteria, including H. pylori (46, 48–51). A preliminary definition of a genome-wide set of CsrA-dependent transcripts in H. pylori was performed previously (43), and our comprehensive data set of T4SS-dependent regulation in the present study showed some overlap. Recent work in E. coli has impressively demonstrated how CsrA acts on a genome-wide scale both as an activator and a repressor (46). We generated and characterized H. pylori *csrA* mutants, which demonstrated that the heptose gene cluster, in addition to known CsrA-regulated transcripts, is under CsrA control and that *csrA* mutants also exert lower proinflammatory activity on human cells. As one signature gene downstream of CsrA, whose activity is governed by fluxes in carbohydrate metabolism (52), we also confirmed *fbp* (encoding fructose-bisphosphatase). The Fbp reaction product, fructose-1,6-bisphosphate, is an upstream modulator of central metabolism and glycolytic flux (glycolysis and tricarboxylic acid [TCA] cycle activation versus gluconeogenesis) in bacteria (53). We found increased *fbp* transcript expression alongside *csrA* after bacterial coincubation with human gastric epithelial cells. The results are suggestive of a global regulatory switch of H. pylori phenotype between ecological conditions (e.g., planktonic versus cell-associated) under which cell-directed heptose transport and other functions are either shut off or switched on.

In conclusion, H. pylori produces a proinflammatory heptose-monophosphate MAMP, most likely β-HMP-1, in addition to β-d-ADP-heptose and HBP. Additionally, heptose biosynthesis pathway activity and HldE expression are regulated strain specifically and are dependent on CagT4SS activity, which entails partial CsrA-dependent regulatory feedback on heptose biosynthesis and global functions. Hence, intracellular heptose metabolite amounts and an active CagT4SS are likely to influence bacterial central metabolism and induce cell contact-dependent heptose production “upon” demand. Consequently, H. pylori, an important model pathogen and paradigm of persistently host-associated bacteria, increases its toolbox of mechanisms to influence and fine-tune its production of cell-active, translocated, proinflammatory carbohydrate metabolites and thereby possibly combines innate immune activation of host cells with metabolic cross-talk.

## MATERIALS AND METHODS

### Bacterial strains and culture conditions of H. pylori strains.

H. pylori bacteria of various wild-type strains and corresponding isogenic allelic exchange mutants in heptose biosynthesis genes, *cagY*, and the *cag*PAI were used as listed with references in Table 1. The mutants were generated as reported previously (11). For novel *cag*PAI deletion mutants in L7 and Su2, the insertion mutations were recreated by the same strategy as previously described (11). The *cag*PAI deletion mutant is a complete deletion, while all single-gene mutants were designed as insertion mutations to retain 3′ and 5′ segments of the genes interrupted by a kanamycin resistance cassette. For routine culture, H. pylori strains were grown on blood agar plates (Oxoid blood agar plates base II) supplemented with 10% horse blood (Oxoid) and the following antibiotics for selective growth of insertion mutants (all purchased from Sigma-Aldrich, USA): amphotericin B (4 mg/L), polymyxin B (2,500 U/L), vancomycin (10 mg/L), trimethoprim (5 mg/L), and kanamycin (optional; 10 mg/L). For cell coincubations, transcriptome analyses, and preparations of lysates, bacteria were freshly grown for 20 to 24 h on plates in anaerobic jars supplemented with Anaerocult C sachets (Merck, USA) at 37°C. A list of strains used in the present study is provided in Table 1.

### H. pylori *csrA* mutant.

The H. pylori *csrA* mutant was generated by inserting a kanamycin cassette (aphA3′-III) into the H. pylori *csrA* gene and cloning it into pUC18 using PCR and *csrA*-specific primers (Table 4). The construction of the *csrA* mutant was performed with initial amplification of *csrA* (HP1442) and *csrA*-flanking regions from H. pylori 26695 genomic DNA and introducing PstI and KpnI restriction sites (resulting in plasmid pCJ2008). Primers HP_1442del_bglII_fw and HP_1442del_bglII_rv (Table 4) were then used to reverse amplify and religate plasmid pCJ2009 from pCJ2008. pCJ2009 contains the restriction site BglII in the center of *csrA*, with a 42-bp partial deletion of the gene. Using the BglII site, the kanamycin cassette (cut BamHI) was inserted, generating plasmid pCJ2010. The resistance cassette insertion in the chromosomal *csrA* locus of H. pylori strain N6 was generated after natural transformation of pCJ2010 by allelic exchange mutagenesis. *csrA* insertion mutants were selected on blood agar containing kanamycin and checked for correct chromosomal insertion-recombination using cloning primers HP_1442_PstI_fw and HP_1442_KpnI_rv and resistance cassette-specific primers.

**TABLE 4:** Primers for cloning

Primer name	Sequence (5′→3′)	Description
HP0858xp_BamHI_fw	TATGGATCCAAAAAAATCTTAGTCATAGGCGATCTGA	Cloning of HP0858 d1 of strain HP26695 in pET28a (His tag in frame); cloning of HP0858 (complete gene) of strain HPN6 in pET28a (His tag in frame)
HP0858_rv5	TATGCGGCCGCTCATTCTAAAGTTTCTAACAGCTTTTCTAAAG	Cloning of HP0858 d1 of strain HP26695 in pET28a (His tag in frame)
HP0858_fw5	TATGGATCCCAAAAAATCGTTTTCACCAATGG	Cloning of HP0858 d2 of strain HP26695 in pET28a (His tag in frame)
HP0858xp_NotI_rv	TATGCGGCCGCTCAATCATTGCATGTCCTTTTAATTTTTTCTA	Cloning of HP0858 d2 of strain HP26695 in pET28a (His tag in frame); cloning of HP0858 (complete gene) of strain HPN6 in pET28a (His tag in frame)
HP1442del_PstI_fw	AAACTGCAGATTATCCAACTTACCGCTTA	Cloning of HP1442 (*csrA*) and flanking regions into pUC18
HP1442del_KpnI_rv	AAAGGTACCGCCATAAGAAGTGGCGTTAG	Cloning of HP1442 (*csrA*) and flanking regions into pUC18
HP1442del_BglII_rv	AAAAGATCTCTTTGCGGCTGAGTATGAGC	Reverse amplification and insertion of restriction site for *aphA3*′-*III* (km)^*a*^ cassette
HP1442del_BglII_fw	AAAAGATCTAGAGGGAGTGTGCGTTTAGG	Reverse amplification and insertion of restriction site for *aphA3*′-*III* (km)^*a*^ cassette

a km, kanamycin; restriction sites are underlined.

### Cultivation of human cells.

The human cell lines AGS (ATCC, CRL-1739; human gastric adenocarcinoma cell line) and MKN28 (JCRB0253; human gastric carcinoma cell line) were routinely cultured in RPMI 1640 medium buffered with 20 mM HEPES and GlutaMAX stable amino acids (Gibco, Thermo Fisher Scientific, USA). Medium was supplemented with 10% fetal calf serum (FCS; PromoCell, Germany). AGS and MKN28 cells were cultured without antibiotics. The cell line HEK-NF-κB_luc (BPS Bioscience, USA; luciferase reporter cell line) was routinely cultured in Dulbecco’s modified Eagle’s medium (DMEM; buffered with 20 mM HEPES) supplemented with GlutaMAX (Gibco, Thermo Fisher Scientific, USA) and 10% FCS (PromoCell, Germany). HEK-NF-κB_luc cells were supplemented for routine growth with 50 μg/mL hygromycin B (Invivogen, USA) in the same culture medium. For infection and coincubation experiments, all cell lines were seeded in medium without antibiotics. Antibiotics were removed from all cell cultures by washing before starting coincubation assays. All cell cultures were grown in a 5% CO_2_ atmosphere incubator and routinely passaged using 0.05% buffered trypsin-EDTA (Gibco, Thermo Fisher Scientific, USA).

### Coculture of cells with live bacteria or bacterial products.

Human cells were cocultured with various H. pylori strains or bacterial lysates. The infection was performed in 6-, 24-, or 96-well plates (Greiner Bio-One, Austria) on subconfluent cell layers (60 to 80% confluence) seeded on the previous day. Sixty minutes before infection, the medium was exchanged to fresh RPMI 1640 (supplemented with 20 mM HEPES, GlutaMAX, and 10% FCS) or fresh DMEM. All bacteria-cell or compound- and lysate-cell coincubation experiments were performed in the absence of antibiotics. For infection of cells with bacteria, H. pylori was harvested after 20 h of growth from fresh blood plates into cell culture medium. The optical density at 600 nm (OD_600_) of this bacterial suspension was measured and adjusted to the respective multiplicities of infection (MOIs; an MOI of 25 was used in most experiments), as indicated in the results and figures. The coincubation was synchronized by centrifugation of the cell culture plates (300 rpm for 5 min at room temperature). For coincubation with enzymatically treated lysates (ETLs) or β-d-ADP-heptose (Invivogen), cell medium was also changed 60 min before the addition of lysate preparation or β-d-ADP-heptose to the cells to fresh medium without antibiotics, and coincubation started with centrifugation of the plate. Coincubation was performed in a 5% CO_2_ atmosphere cell incubator for different incubation periods (indicated in text and figures). Samples were harvested after taking off the supernatant carefully (for ELISA, IL-8 secretion), either by scraping the cells from the bottom of the plate (for RNA isolation) or by adding luminescence substrate to the cells and medium (for NF-κB luciferase quantitation). If not otherwise indicated, cells were coincubated with bacteria or metabolite preparations for 4 h for measuring IL-8 secretion or for performing luminescence reporter assays.

### RNA isolation of human and bacterial samples.

RNA was isolated from human cells, bacteria, or bacteria-cell coincubation samples. Human cells (cocultured) were harvested from 6-well plates (Greiner Bio-One, Austria) or small petri dishes (diameter of 3 cm) by scraping the cells from the surface using a rubber policeman. H. pylori strains were harvested by resuspending bacteria from plates after approximately 20 h of growth. Human coincubated or bacterial samples were centrifuged, and pellets were snap-frozen immediately in liquid nitrogen or on dry ice and stored at −80°C until RNA preparation. From pellets, total RNA was isolated using an RNeasy minikit (Qiagen, Germany) following the manufacturer’s instructions after mechanical lysis of the samples in a FastPrep bead beater (MP Biomedicals, Inc., USA) at 5 MHz for 45 s using lysing matrix B (for bacterial samples; MP Biomedicals). Isolated RNA was treated with DNase using a TURBO DNase cleanup kit (Ambion-Invitrogen, USA). Sufficient RNA quality and purity were ensured by photometric measurement, gel electrophoresis, and RNA ScreenTape analysis in a Tape Station (Agilent, USA) using high-sensitivity RNA tapes and by the amplification of control genes (H. pylori 16S rRNA genes for control of RNA purity). Total RNA was then used for genome-wide RNA-sequencing (RNA-seq) or further processed for cDNA generation and performance of RT-qPCR.

### cDNA synthesis and RT-qPCR.

cDNA was synthesized from 1 μg of total bacterial RNA using Superscript III reverse transcriptase (Invitrogen, USA), RNase-Out (Invitrogen, USA), and random nonamer primers. Reverse transcription was performed from 1 μg of total RNA. All reagents were purchased from Invitrogen (USA). Sufficient quality of cDNA was ensured by control PCRs (amplification of 16S for bacterial cDNA).

RT-qPCR was routinely performed on 0.5 μL of cDNA in a CFX96 real-time PCR cycling machine (Bio-Rad, USA) using H. pylori gene-specific primers (synthesized at Metabion) (Table 5) and 2× SYBR Green master mix (Qiagen, Hilden, Germany). Transcript quantification was always performed in triplicate, with gene-specific standards for absolute amount quantification, and performed in parallel for each transcript using the following protocol: 95°C for 10 min; 95°C for 30 s, 55°C for 30 s, and 72°C for 30 s for 40 cycles; and a melting curve of 60°C to 95°C with an increment of 0.5°C for 5 s. Results were equalized to 1 μL of cDNA and normalized to transcript amounts of the H. pylori 16S gene using a correction factor respective to the mock reference but maintaining the absolute values (pg/μL) for all final transcript amounts. MiQE standards for the RT-qPCR method were applied as detailed in reference 37. Statistics for Fig. 1C to F are provided in Table S1 in the supplemental material. Each figure shows different biological experiments, which explains the slight variation of absolute transcript quantities. Different experiments can have slightly different starting quantities/transcript amounts of certain transcripts due to biological variation.

**TABLE 5:** List of gene-specific primers used for RT-qPCR

Primer name	Sequence (5′→3′)	Description
HP0857_qPCR_fw1	TAGCCCATAAGGAAGCGTTA	Amplification of gene HP0857
HP0857_qPCR_rv1	GTCAATTCAGCGGCAAAATG	Amplification of gene HP0857
HP0859_qPCR_fw1	GCATTTTGATTATTTGTTCCACC	Amplification of gene HP0859
HP0859_qPCR_rv1	CGCTGAAGAAGCGTAAATCA	Amplification of gene HP0859
HP0860_qPCR_fw1	CAGAGACGGCATTATCAATATTG	Amplification of gene HP0860
HP0860_qPCR_rv1	GATTGGTTGGTGATTAAAAGCA	Amplification of gene HP0860
HP0858_qPCR_fw1	CGAGTTTAGAAGAAATCGCT	Amplification of gene HP0858
HP0858_qPCR_rv1	CCTAAAGCTTTAGCCTTTTGC	Amplification of gene HP0858
HP1385_qPCR_fw1	TAAAGCGGATTTAGCCCTAG	Amplification of gene HP1385
HP1385_qPCR_rv1	CATAAGCGATCAAATAAGAGCC	Amplification of gene HP1385
HP1442_qPCR_fw1	GAAGGGATTGTCATTGATGATAAC	Amplification of gene HP1442
HP1442_qPCR_rv1	ACAATGGCCTCTTTGAGTTC	Amplification of gene HP1442

### RNA-seq and data analysis.

RNA-seq and transcriptome analyses were performed as previously described (12). Briefly, paired, trimmed, and quality-filtered fastq files with an average read length of 150 bp obtained from the Illumina NextSeq2500 platform were processed using the CLC Genomics Workbench version 20.0 (Qiagen, Germany). Paired reads were downsampled for each sample to 2,000,000 and mapped against the H. pylori reference genome 26695 (accession number AE000511, NCBI database) (53). Reads were aligned as previously described (12), and gene expression levels were quantitated as reads per kilobase per million (RPKM) values normalized to the gene length and read number. RPKM values were then used for calculating differential expression between samples using the CLC Genomic Workbench RNA-Seq analysis workflow. See Table S2 for full transcriptome results and differentially expressed genes. Full results are accessible on NCBI’s Gene Expression Omnibus (GEO) under accession number GSE227450.

### ETLs and water lysates.

Bacterial lysates were prepared from H. pylori harvested into sterile cell culture 1× phosphate-buffered saline (PBS; Gibco, Thermo Fisher Scientific, USA) from blood agar plates after 20 h of growth. The bacterial suspension, quantified by OD_600_ measurement, was centrifuged, and the pellets were stored at −20°C. Briefly, for lysate preparation, the frozen pellet was resuspended in an appropriate volume (1 mL) of 1× PBS and adjusted to an OD_600_ of 2 (per mL). The suspension was boiled for 10 min in a water bath (99°C) and centrifuged. The supernatant was sterile filtered (0.22-μm-pore filter; Merck Millipore, Germany) and stored at −20°C if not further processed immediately. Further processing of the samples was performed either by enzymatic treatment (generation of ultrapure ETLs), as described previously (11), or by methanol precipitation. For NMR measurements (less-pure lysate preparations, which still contain, for instance, residual DNA), lysates of an OD_600_ of 20, 10, or 5 in 1 mL were prepared in PBS as described above and boiled for 20 min. These lysates were then additionally treated for protein precipitation with 3 volumes of ultrapure methanol (Sigma-Aldrich, USA). Ultimately, samples were vortexed and centrifuged to precipitate proteins and to recover protein-free supernatants, which were again sterile filtered. Ultrapure ETL samples used for mass spectrometry were additionally mixed with methanol and acetonitrile (1:2:2 [vol/vol/vol] final mixture).

### Cytokine measurement from cell supernatants.

IL-8 secretion from human cells to the supernatant was quantified by performing a human IL-8 ELISA, according to the manufacturer’s protocol (BD OptEIA, 555244), on pretested sample dilutions. Colorimetric signal detection was performed using a Clariostar multiwell plate-reading machine (BMG Labtech).

### Luciferase quantitation in human reporter cells.

Briefly, firefly luciferase signal of HEK-NF-κB_luc cells was determined using a Steady Glo/Bright Glo luciferase assay (Promega, USA), as previously described (12). Luciferase quantification was regularly performed in 96-well F-bottom plates (Greiner BioOne, Austria, 655180) using 50 μL of total sample volume. After 4 h of coincubation with live bacteria, bacterial lysates, β-d-ADP-heptose, or other metabolites (as indicated in the Results and figures), equal volumes of the luciferase lysing and detection buffer (Promega) were added to each well. The reaction was allowed to incubate for 10 min for cell lysis with shaking, followed by luminescence measurement in a Victor Nivo Multimode microplate reader (PerkinElmer, USA) at the following settings: shaking for 3 s, no filter, 1-s photon counting. All conditions were analyzed in duplicate or triplicate.

### SDS-PAGE and Western blotting for protein detection and quantification.

Bacterial samples were prepared by harvesting bacteria directly from the plate, after centrifugation, into 1× PBS, followed by ultrasonication (Branson sonifier, 2 × 1 min at power setting 5) and subsequent separation of soluble and insoluble fractions by centrifugation (10,000 × *g*, 20 min, 4°C). The concentration of protein in each sample was determined by bicinchoninic acid (BCA) assay (Pierce, Thermo Fisher Scientific, USA) using a Clariostar multiwell reader for final colorimetric readings. Regularly, 10 μg of protein (equalized amounts for all samples) was loaded onto 11.8 to 14% SDS gels and run at 100 V constant voltage in Laemmli buffer supplemented with 0.1% SDS. Blotting was performed onto BA85 nitrocellulose membranes (Schleicher & Schuell) in Towbin buffer for 2 h at 300 mA. Blotted membranes were blocked using 5% skim milk (Sigma-Aldrich, USA or Bio-Rad, USA) or 1 to 5% bovine serum albumin (BSA) in Tris-buffered saline (TBS) buffer containing 0.1% Tween (TBS-T), according to antibody specifications. Specific antibody (Table 6) incubations were performed for 1 h at ambient temperature or overnight at 4°C. As secondary antibody, goat anti-rabbit or goat anti-mouse antibody coupled to horseradish peroxidase (HRP; Jackson Immuno Laboratories) was used at a dilution of 1:10,000. Signal was detected using Immobilon HRP chemiluminescence substrate detection reagent (Merck Millipore, Germany) and imaged in a chemiluminescent imager (Bio-Rad, USA).

**TABLE 6:** Specific antisera and antibodies used in this study

Antigen	Source species	Reference
HldE	Rabbit	This study
FlhA	Rabbit	61

### DNA cloning methods for protein expression.

For expression of heptose biosynthesis enzymes, genes HP0857 (*gmhA*), HP0858 (*hldE*), and HP0860 (*gmhB*) were cloned from PCR products generated from genomic DNA of the respective H. pylori wild-type strains, 26695a and N6, using BamHI and NotI (New England BioLabs [NEB], USA) as full-length constructs in pET28a(+) (EMD Biosciences, Novagen, Germany). The two strains were selected for their diversity in *hldE* sequence. To express the two domains of the bifunctional enzyme HldE (HP0858), the first (amino acids 1 to 325) and last segments of the gene (amino acids 328 to 461) were cloned separately into pET28a(+) using the same restriction enzymes. Gene constructs were located behind a 6× N-terminal His tag, separable from the enzyme using the tobacco etch virus (TEV) protease cleavage site between construct and His tag. The plasmids confer kanamycin resistance allowing selection of clones. Clones were checked by restriction analysis and Sanger sequencing of the complete inserts. The following were the resulting plasmids: HP0858/*hldE* (strain 26695) is pCJ1628, HP0858/*hldE* (strain N6) is pCJ2004, HP0857/*gmhA* (26695) is pCJ1627, HP0860/*gmhB* (26695) is pCJ1630, HP0858/*hldE* (26695) d1 is pCJ2002, and HP0858/*hldE* d2 is pCJ2003. The primers used for cloning are listed in Table 4.

### HEK cell transfection for metabolite activity testing (ETLs).

HEK-NF-κB_luc reporter cells (BPS Bioscience, USA) were transfected with bacterial ETLs or *in vitro*-synthesized heptose metabolites using Lipofectamine 2000 transfection agent (Invitrogen). Transfection was performed in 96-well plates, and cells were seeded to 3 × 10^4^ cells in 50 μL of DMEM (containing 10% FCS) per well approximately 20 h before transfection. Medium was exchanged to 25 μL of Opti-MEM (containing 5% FCS) 1 h before the addition of 25 μL of transfection agent containing 4 μL of ETL or heptose metabolites, 0.5 μL of Lipofectamine 2000, and 20.5 μL of Opti-MEM per well. Transfected cells were subsequently incubated at standard culture conditions; after 4 h of incubation, luciferase substrate buffer (SteadyGlo, Promega) was added for cell lysis, and luminescence was detected as described above.

### Protein expression and purification from E. coli.

Expression of heptose biosynthesis enzymes from inducible expression plasmids was performed in Luria-Bertani (LB) broth (Lennox [Oxoid]) supplemented with 50 μg/μL kanamycin (Sigma, USA) from expression cultures set up in E. coli Rosetta pLysS (Novagen/Merck, Germany) inoculated from overnight culture to a starting OD_600_ of 0.1. At an OD_600_ of 0.5 to 0.8, expression cultures were induced with 0.1 to 0.5 mM isopropyl-β-d-thiogalactopyranoside (IPTG) and grown to express protein for 4.5 h at 30°C (with 175 rpm shaking). Bacterial pellets were harvested by centrifugation (10,000 × *g*, 10 min, 4°C) and stored at −20°C before further purification. Proteins were purified from the soluble (native) fraction after the lysis of bacterial pellets by ultrasonication using a 1-mL nickel-nitrilotriacetic acid (Ni-NTA) fast protein liquid chromatography (FPLC) affinity chromatography column (Macherey & Nagel, Germany) coupled to an Äkta Purifier or Prime system (Cytiva/GE Healthcare, USA) at 20°C (room temperature) or 4°C (the latter for N6 HldE and 26695 GmhB). After loading the column, nonspecific impurities were removed by extensively washing the column with purification buffer (50 mM Tris-HCl [pH 7.5], 300 mM NaCl, and 1 mM dithiothreitol [DTT]) and high-salt purification buffer (1 M NaCl added to the previous), followed by equilibration in purification buffer. Gradient elution was performed by increasing the imidazole concentration of the purification buffer/lysis buffer gradually from 0 to 500 mM. High-protein-content elution fractions were pooled, dialyzed into buffer without imidazole, and further characterized, and protein purity and quantity were analyzed in SDS gels using appropriate reference protein loadings.

### Generation of an anti-HldE antiserum.

The antiserum was raised in rabbits against overexpressed, column-purified, full-length H. pylori HldE protein (cloned from strain 26695).

### *In vitro* reconstitution of the H. pylori heptose biosynthesis pathway.

All three central enzymes of the pathway (GmhA, HldE, and GmhB), cloned from H. pylori into the pET28a vector, were overexpressed and purified from E. coli by Ni^2+^-affinity chromatography (see earlier). All proteins were purified from the soluble fraction in native form. *In vitro* reconstitution of the biosynthetic pathway was performed as follows: about 5 to 10 nmol (1 to 2 μg) of each protein were combined into a 50-μL reaction in an inert reaction buffer without any phosphate and with or without the substrate seduheptulose-7-phosphate (S7-P; buffer containing 20 mM HEPES [pH 7.5], 5.8 mM ATP, 20 mM KCl, and 10 mM MgCl_2_); reaction mixtures were incubated overnight (approximately 18 h) at 37°C. After heat inactivation (HI), this material was directly used for all cell coincubations. For NMR analysis, the samples were boiled to inactivate any proteins or enzymes and precipitated with methanol (1:3 [vol/vol]; 1 volume reaction mixture to 3 volumes methanol) to remove denatured protein. After protein removal by centrifugation, the supernatant containing the metabolite reaction products was then concentrated to dryness by a flow of gaseous nitrogen. For mass spectrometry, the reaction mixtures were scaled up to 300-μL volumes.

### NMR and mass spectrometry analysis of bacterial heptose metabolites and reaction products.

**(i) Reference ADP-heptose for NMR analysis.** Fifty microliters of each commercial reagent, β-d-ADP-heptose (2 mM; Invivogen, France), and β-l-ADP-heptose (J&K, China), was dried under a stream of nitrogen and dissolved in 120 μL of heavy water (D_2_O). These were used as references for the chemical identity of H. pylori-produced ADP-heptose isomers.

**(a) Sample preparation.** The following samples were prepared for NMR analysis of heptose metabolites (compare also Table 3): 2, control sample containing 20 mM HEPES buffer at pH 7.5 (20 mM KCl, 10 mM MgCl_2_, and 6 mM NaATP) and H. pylori purified pathway enzymes GmhA, HldE, and GmhB but no pathway substrate; 6, HldE and substrate seduheptulose-7-phosphate (S7-P; 2 mM; Sigma-Aldrich), putative product is S7-P; 11, enzymes GmhA and HldE and substrate S7-P (2 mM, Sigma-Aldrich), putative product is β-d-heptose-1,7-bisphosphate (d-glycero-β-d-manno-heptose-1,7-biphosphate [β-d-HBP]); 9, enzymes GmhA, HldE, and GmhB and substrate S7-P (2 mM), putative product is ADP-heptose. All samples were incubated for metabolite biosynthesis at 37°C overnight and subsequently heat inactivated at 95°C for 20 min. Afterward, all samples were treated with methanol (3:1 methanol:samples) for protein precipitation and pelleted by centrifugation. Each of the supernatants was dried under a stream of N_2_ at about 40°C for 30 min, and the remaining residue was dissolved in 120 μL of D_2_O. Each of the solutions was carefully filled into a Bruker NMR Match microtube.

**(ii) NMR spectroscopy for metabolite identification and characterization.** All NMR spectra were recorded with a Bruker AVANCE 500 MHz spectrometer equipped with an solid electrolyte interface (SEI) probe using TopSpin version 3.5 (Bruker Biospin, GmbH, Rheinstetten, Germany). ^1^H NMR spectra were recorded with the Bruker pulse program “noesygppr1d” for suppression of the water signal during the relaxation period, applying a narrow saturation pulse with a line width of about 25 Hz. The parameters were ns = 64 or 256, ds = 8 s, TE = 25°C, aq = 2.73 s, td = 32,768, sw = 12.0 ppm, and p1 = 8.20 μs. ^1^H,^1^H-COSY spectra with water suppression were recorded with the Bruker pulse program “cosygpprqf” with ns = 4 or 64, ds = 8 s, TE = 25°C, aq = 0.10 s, td = 1,024 (f2), 256 (f1), sw = 10.00 ppm, and p1 = 8.20 μs. Data were processed with MestreNova version 14.2.0 (Mestrelab Research, Santiago de Compostela, Spain). The flame ionization detections (FIDs) were zero filled and multiplied by a mild Gaussian function before Fourier transformation. Under these conditions, the detection limit for ADP-heptose was approximately 27 μg of dissolved ADP-heptose, that is, 361.8 μM (applying 128 scans in the one-dimensional experiment). The ^1^H-NMR spectrum of 830 μM ADP-heptose is shown in Fig. 6E, 6F. Results for other reference samples are documented in Fig. 6A through 6D. With the help of ^1^H,^1^H-COSY and by comparing to previously published data (42, 54), the detected signals could be clearly assigned to protons of the adenine unit, the ribose unit, and the heptose unit. Further on, characteristic CH_3_ signals at 1.21 ppm and CH_2_ signals at 3.14 ppm belonging to trimethylamine were detected for the reference compound. Notably, triethylamine is often used during the chemical synthesis of ADP-heptose as a protection reagent and is present as the counter ion in the reference sample (8). Chemical shifts, multiplicity, coupling constants, and correlations observed in the ^1^H,^1^H-COSY spectrum for substrates, reference compounds (S7-P, β-d-ADP-heptose, and β-l-ADP-heptose), and products are presented in Table S3.

**(iii) Mass spectrometry (LC-ESI MS/MS) of heptose metabolites.** Pure reagents, *in vitro* reaction mixtures, or cell extracts were analyzed on an Agilent 6495 triple quadrupole mass quadrupole mass spectrometer equipped with an ESI ion source and coupled to an Agilent 1290 Infinity II ultrahigh performance liquid chromatographer (UHPLC; both Agilent Technologies). Chromatographic separation of ADP-heptose was achieved with an Acquity ultraperformance liquid chromatography (UPLC) BEH amide (1.7 μm, 2.1 × 100 mm) column (Waters). Solvent A consisted of water with ammonium formate (10 mM) and formic acid (0.1% vol/vol). Solvent B consisted of acetonitrile with formic acid (0.1% vol/vol). The LC gradient was as follows: 0 min 90% B, 7 min 40% B, 8 min 40% B, 8.5 min 90% B, and 12 min 90% B (flow rate of 0.1 mL/min). The heptose-phosphates were separated by a SeQuant ZIC-pHILIC (5 μm, 150 × 2.1 mm) column (Merck). Solvent A consisted of water with ammonium carbonate (10 mM) and ammonium hydroxide (0.2%). Solvent B consisted of acetonitrile. The LC gradient was as follows: 0 min 90% B, 12 min 40% B, 14 min 40% B, 15 min 90% B, and 18 min 90% B (flow rate of 0.2 mL/min). Three microliters was injected per sample. The settings of the ESI source were 200°C source gas temperature, 14 L/min drying gas, and 24 lb/in^2^ nebulizer pressure. The sheath gas temperature was 250°C, and the flow was 11 L/min. The electrospray nozzle was set to 500 V, and capillary voltage was 2,500 V. ADP-heptose was analyzed in positive ion mode with a transition from 620 *m*/*z* to 428 *m*/*z* (collision energy of 10 keV and a dwell time of 100 ms). Heptose-bisphosphate was analyzed in positive ion mode with a transition from 371 *m*/*z* to 273 *m*/*z* (collision energy of 10 keV and dwell time of 100 ms). Heptose-monophosphates were analyzed in negative ion mode with a transition from 289 *m*/*z* to 79 *m*/*z* (collision energy of 40 keV and dwell time of 100 ms). Raw data were converted into text files using MSConvert (55). Data analysis was performed with a customized MATLAB script. Bioblanks were also measured by spiking the reference compounds into bacterial lysates to determine possible shifts in retention times.

### Data availability.

Full RNA-seq results are accessible on NCBI’s GEO under accession number GSE227450.
